# Potentiating adoptive cell therapy using synthetic IL-9 receptors

**DOI:** 10.1038/s41586-022-04801-2

**Published:** 2022-06-08

**Authors:** Anusha Kalbasi, Mikko Siurala, Leon L. Su, Mito Tariveranmoshabad, Lora K. Picton, Pranali Ravikumar, Peng Li, Jian-Xin Lin, Helena Escuin-Ordinas, Tong Da, Sarah V. Kremer, Amy L. Sun, Sofia Castelli, Sangya Agarwal, John Scholler, Decheng Song, Philipp C. Rommel, Enrico Radaelli, Regina M. Young, Warren J. Leonard, Antoni Ribas, Carl H. June, K. Christopher Garcia

**Affiliations:** 1 Department of Radiation Oncology, David Geffen School of Medicine and Jonsson Comprehensive Cancer Center, University of California, Los Angeles, Los Angeles, CA USA; 2 Parker Institute for Cancer Immunotherapy, San Francisco, CA USA; 3 Center for Cellular Immunotherapies, Perelman School of Medicine, University of Pennsylvania, Philadelphia, PA USA; 4 Departments of Molecular and Cellular Physiology and Structural Biology, Stanford University School of Medicine, Stanford, CA USA; 5 Laboratory of Molecular Immunology and the Immunology Center, National Heart, Lung, and Blood Institute, National Institutes of Health, Bethesda, MD USA; 6 Division of Hematology/Oncology, Department of Medicine, David Geffen School of Medicine and Jonsson Comprehensive Cancer Center, University of California, Los Angeles, Los Angeles, CA USA; 7 Penn Vet Comparative Pathology Core, Department of Pathobiology, University of Pennsylvania, Philadelphia, PA USA; 8 Stanford Cancer Institute, Stanford University School of Medicine, Stanford, CA USA; 9 Howard Hughes Medical Institute, Stanford University School of Medicine, Stanford, CA USA

**Keywords:** Synthetic biology, Immunotherapy, Cancer therapy, Interleukins

## Abstract

Synthetic receptor signalling has the potential to endow adoptively transferred T cells with new functions that overcome major barriers in the treatment of solid tumours, including the need for conditioning chemotherapy^1,2^. Here we designed chimeric receptors that have an orthogonal IL-2 receptor extracellular domain (ECD) fused with the intracellular domain (ICD) of receptors for common γ-chain (γ_c_) cytokines IL-4, IL-7, IL-9 and IL-21 such that the orthogonal IL-2 cytokine elicits the corresponding γ_c_ cytokine signal. Of these, T cells that signal through the chimeric orthogonal IL-2Rβ-ECD–IL-9R-ICD (o9R) are distinguished by the concomitant activation of STAT1, STAT3 and STAT5 and assume characteristics of stem cell memory and effector T cells. Compared to o2R T cells, o9R T cells have superior anti-tumour efficacy in two recalcitrant syngeneic mouse solid tumour models of melanoma and pancreatic cancer and are effective even in the absence of conditioning lymphodepletion. Therefore, by repurposing IL-9R signalling using a chimeric orthogonal cytokine receptor, T cells gain new functions, and this results in improved anti-tumour activity for hard-to-treat solid tumours.

## Abstract (web summary)

Synthetic chimeric orthogonal IL-2 receptors that incorporate the intracellular domain of receptors for other γ-chain cytokines such as IL-9 can reroute orthogonal signalling and alter the phenotype of T cells to improve anti-tumour responses.

## Main

Therapies that use adoptively transferred genetically engineered T cells have shown substantial anti-tumour activity in patients with haematopoietic malignancies, but have limited benefit in patients with solid tumours. One major limitation is the poor in vivo expansion and persistence of adoptively transferred T cells, which necessitates lymphodepleting conditioning chemotherapy—a toxic regimen that limits patient eligibility. Even those T cells that expand and persist become terminally differentiated and dysfunctional^3,4^. Synthetic cytokine receptor signalling could reprogram T cells with a stem-like phenotype that can overcome these limitations and exhibit improved anti-tumour activity in mouse models and humans^5,6^. Existing therapeutic manipulations to select or expand stem-like T cells^7,8^ can only be used in the cell manufacturing phase as they lack the specificity for adoptively transferred cells in vivo.

**Fig. 1:**
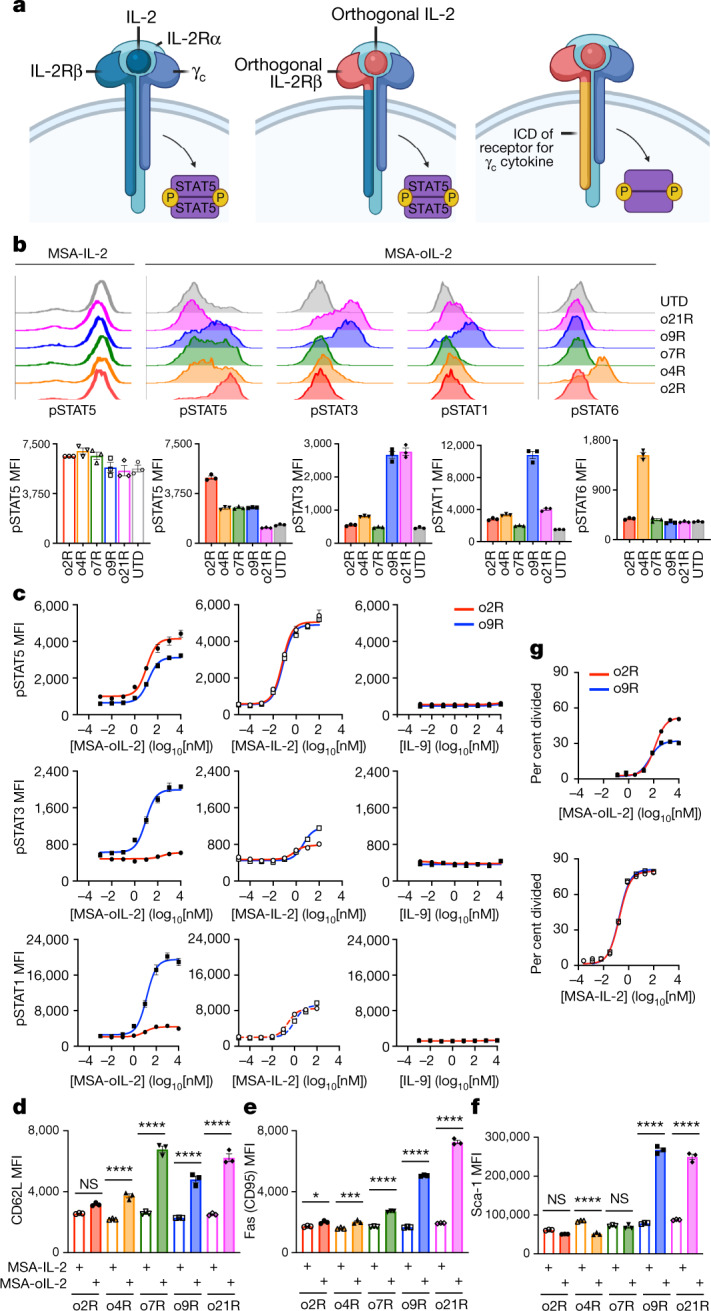
A chimeric orthogonal IL-2 receptor reveals properties of IL-9R signalling in T cells. **a**, Schematic of wild-type IL-2Rβ, orthogonal IL-2Rβ or γ_c_ family chimeric orthogonal receptor complexes (created with Biorender.com). **b**, Representative histogram and quantification of pSTAT signalling in chimeric-orthogonal-receptor-expressing (YFP^+^) or untransduced (UTD) T cells stimulated with MSA-IL-2 (100 nM) (unfilled colour) or MSA-oIL-2 (5 μM) (filled colour) for 20 min. Data are shown as mean fluorescence intensity (MFI). **c**, Dose–response curves of pSTAT signalling in YFP^+^ o2R (red) or o9R (blue) transduced T cells stimulated with MSA-oIL-2, MSA-IL-2 or IL-9 for 20 min. **d**–**f**, Surface marker levels of CD62L (**c**), Fas (CD95) (**e**) and Sca-1 (**f**) of chimeric-orthogonal-receptor-expressing T cells cultured with MSA-IL-2 (100 nM) or MSA-oIL-2 (5 μM) for two days. NS, not significant; *P < 0.05, ***P < 0.001, ****P < 0.0001 (ANOVA). **g**, Dose–response curves of YFP^+^ o2R or o9R cells that have undergone at least one division after four days of culture in MSA-oIL-2 or MSA-IL-2. Data are shown as the percentage divided. Data are mean ± s.e.m. with *n* = 3 biological replicates, unless stated otherwise. Source data

An orthogonal cytokine receptor is a mutant form of the native cytokine receptor that selectively binds to a mutant form of the native cytokine. This has been demonstrated with the orthogonal mouse IL-2 cytokine–receptor pair (oIL-2 and o2R), which allows selective in vivo modulation of adoptively transferred T cells for cancer immunotherapy^9^. The orthogonal mouse IL-2 receptor (o2R) consists of the IL-2Rβ chain with a modified ECD that selectively binds oIL-2, but not wild-type IL-2 (Fig. 1a). Likewise, oIL-2 cannot bind the wild-type IL-2 receptor. To signal, both the orthogonal and the wild-type IL-2 receptor cooperate with the wild-type γ_c_.

Using the modular nature of the oIL-2 system, we wished to investigate the therapeutic potential of other members of the γ_c_ cytokine receptor family^10–12^. By replacing the ICD of o2R with the ICD of receptors for the γ_c_ cytokines IL-4, IL-7, IL-9 and IL-21, we created chimeric orthogonal receptors that contain the mouse oIL-2-binding ECD fused to the ICD of each γ_c_ cytokine family receptor (Fig. 1a and Supplementary Table 1). For our studies we selected oIL-2 clone 3A10 as the receptor ligand due to its superior orthogonality^9^.  

**Extended Data Fig. 1:**
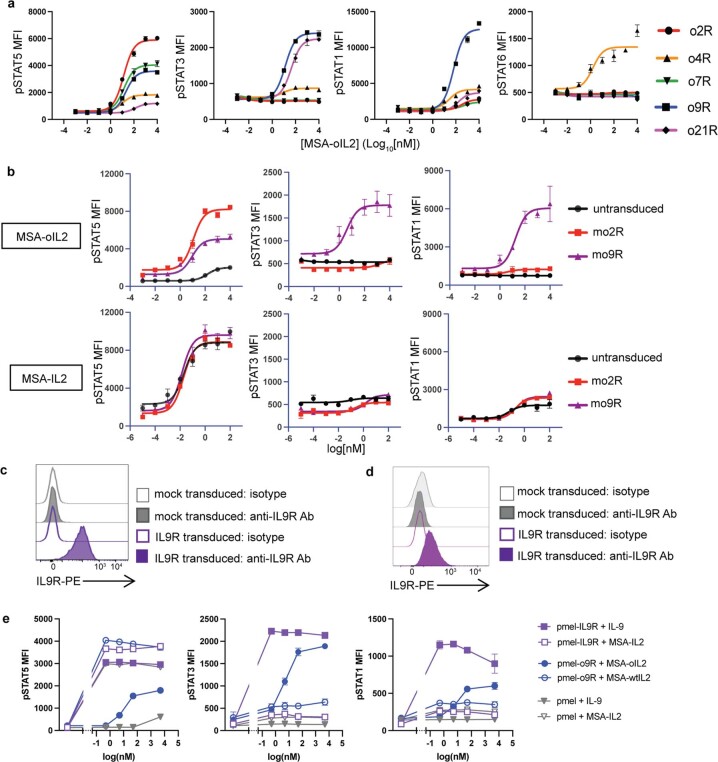
Mechanisms of o9R signalling. **a**, pSTAT-1, -3, -5 and -6 signalling dose response curves of orthoIL-2Rβ ICD chimeric receptor expressing T cells stimulated with MSA-oIL2 for 20’. **b**, pSTAT-1, -3, and -5 signalling dose response of untransduced or o2R or o9R expressing T cells stimulated with MSA-oIL2 or MSA-IL2 for 20’. **c**,**d**, IL-9R expression on mock transduced or IL-9R transduced T cells from C57BL6 (**c**) or pmel TCR transgenic mice (**d**). **e**, Dose–response curves of STAT1, STAT3 and STAT5 phosphorylation in pmel T cells transduced with native IL-9R (pmel-IL-9R), o9R (pmel-o9R), or mock transduced (pmel) and stimulated with IL-9, MSA-IL2, or MSA-oIL2. Data are shown as mean fluorescence ± SEM, n = 3 biological replicates unless otherwise stated. Source data

Stimulation of chimeric orthogonal receptors with mouse serum albumin-bound oIL-2 (MSA-oIL-2, clone 3A10; Supplementary Table 2) resulted in patterns of phospho-STAT signalling that are consistent with known signalling through each respective γ_c_ cytokine^10,13^ (Fig. 1b and Extended Data Fig. 1a). Signalling through o9R resulted in potent phosphorylation of STAT1, STAT3 and STAT5—a distinct profile that is consistent with known signalling through the wild-type IL-9 receptor^14^ (Fig. 1b and Extended Data Fig. 1a). o9R signalling was dose-dependent and specific to MSA-oIL-2 (Fig. 1c). Furthermore, expression of o2R or o9R did not affect wild-type IL-2-induced STAT signalling (Extended Data Fig. 1b).

IL-9R is a less-studied member of the γ_c_ cytokine receptor family that is naturally expressed by mast cells, memory B cells, innate lymphoid cells and haematopoietic progenitors^13,15–20^. Although T cell subsets that produce IL-9 have been described^21–25^, the effects of IL-9R signalling on T cells are not well characterized^26–31^. For example, naive T cells are insensitive to IL-9 and T cell development is unimpaired in IL-9-deficient mice, which suggests that IL-9 is not a critical natural cytokine in T cell biology^18,29,32^. We found that activated mouse T cells did not support IL-9 signalling (Fig. 1c) owing to the absence of IL-9R expression (Extended Data Fig. 1c,d), which underscores the unorthodoxy of o9R signalling in these cells^27,33,34^. o9R signalling is a bona fide mimic of wild-type IL-9R signalling, as IL-9 treatment of mouse T cells transduced with the wild-type IL-9R resulted in a similar pattern of STAT1, STAT3 and STAT5 phosphorylation (Extended Data Fig. 1e).

**Extended Data Fig. 2:**
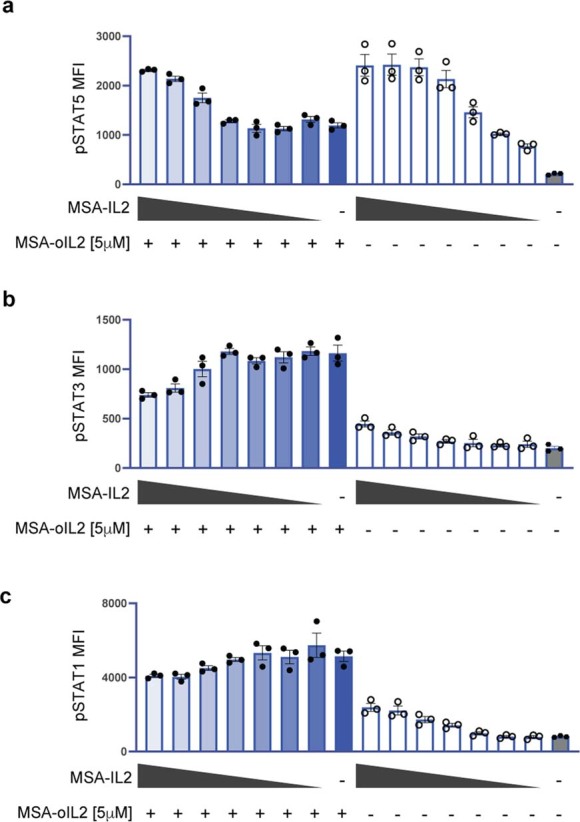
Competition between o9R and wild-type IL-2 signalling. **a**–**c**, pSTAT5 (**a**), pSTAT3 (**b**), and pSTAT1 (**c**) signalling in o9R expressing C57BL/6 T cells treated with 10-fold dose titration of MSA-IL2 (starting at 100nM) in the absence (open) or presence of MSA-oIL2 [5 μM] (filled) for 20’. Data shown as mean fluorescence ± SEM, n = 3 biological replicates, YFP(+) gated. Source data

As o9R cells and wild-type IL-2Rβ both use γ_c_ signalling, we evaluated the competition between native and orthogonal signalling. Co-exposure to saturating doses of MSA-oIL-2 did not affect peak STAT5 phosphorylation by MSA-IL-2 in o9R T cells (Extended Data Fig. 2a). And although increasing doses of MSA-IL-2 partially mitigate MSA-oIL-2 induction of pSTAT1 and pSTAT3 in o9R T cells, likely due to the lower potency of the MSA-oIL-2 3A10 clone, the signalling program of o9R T cells remained active (Extended Data Fig. 2b,c).

**Extended Data Fig. 3:**
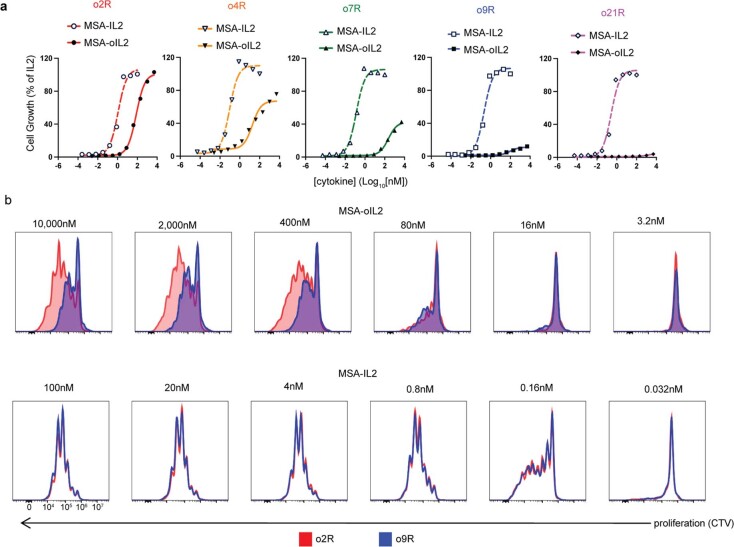
Proliferative effects of chimeric orthogonal receptors. **a**, Dose titration in vitro proliferation curves of o2R and orthogonal chimeric receptor (o4R, o7R, o9R, o21R) expressing T cells cultured for four days in MSA-IL2 (open symbol) or MSA-orthoIL2 (filled symbol). Data represent total live CD8+YFP(+) cells normalized to the maximal growth of each different chimeric receptor expressing cell cultured in MSA-IL2. Data represents mean of n = 2 biological replicates. **b**, In vitro proliferation of CTV-labelled T cells transduced with o2R (red) or o9R (blue) cultured for four days in MSA-oIL2 (filled curves) or MSA-IL2 (unfilled curves). YFP(+) gated; one of three representative plots shown. Source data

We observed that T cells signalling through o9R (as well as o21R) enriched for a CD62L^+^ population and higher expression of Fas (CD95) and Sca-1 consistent with a T_SCM_ phenotype, a subset known for its superior anti-tumour activity in adoptive cell therapy (ACT)^6,35–37^ (Fig. 1d–f). o9R T cells also proliferated less than o2R-expressing T cells (Fig. 1g and Extended Data Fig. 3a,b). Among orthogonal receptors, only o21R resulted in less proliferation than o9R (Extended Data Fig. 3a).

**Fig. 2:**
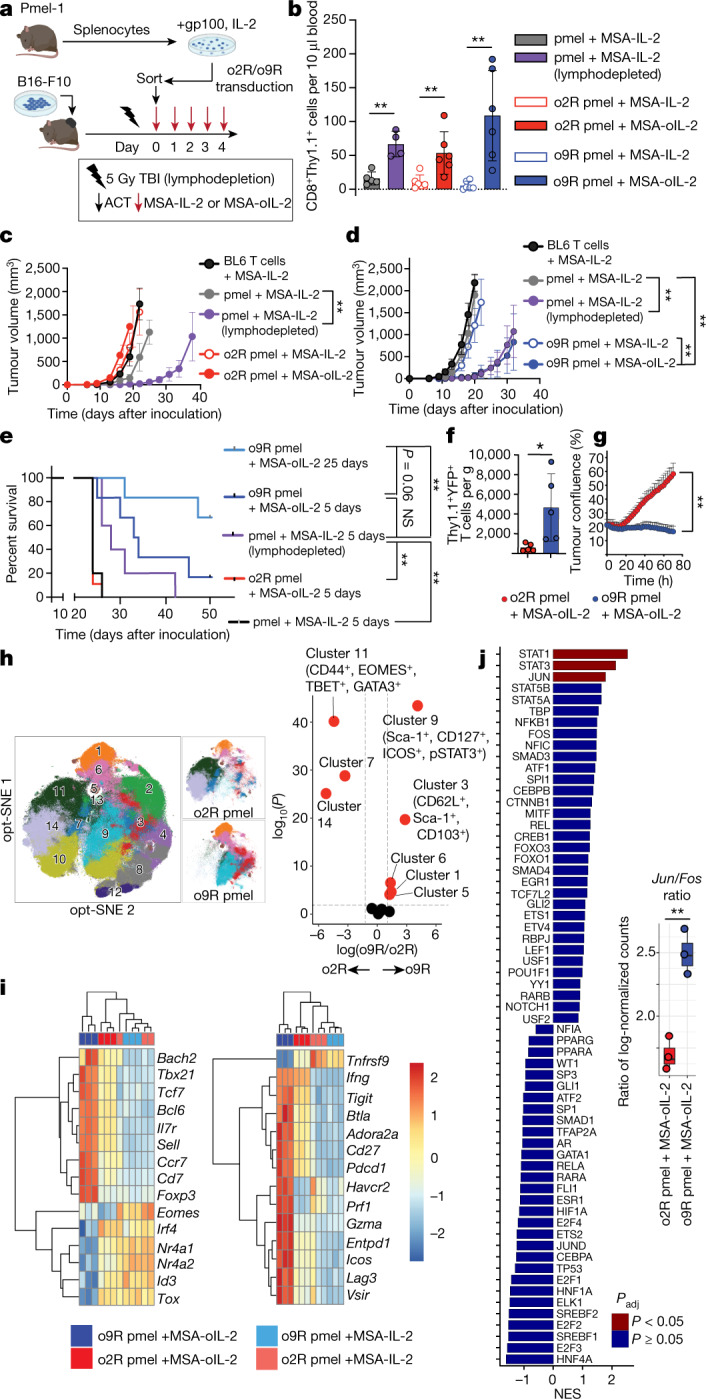
o9R signalling endows pmel T cells with anti-tumour efficacy in the absence of lymphodepletion. **a**, Schematic. B16-F10 melanoma-bearing mice underwent ACT and treatment with MSA-IL-2 or MSA-oIL-2 (2.5 × 10^4^ units per day, intraperitoneal) for 5 days. Mice were not lymphodepleted unless noted. TBI, total body irradiation. **b**, Peripheral blood quantification of pmel T cells seven days after ACT (*n* = 6 mice per group, except where noted in the Methods). **P < 0.01 (unpaired *t*-test). **c**,**d**, Tumour growth (mean ± s.e.m., *n* = 6 mice per group, except where noted in the Methods) after treatment with ACT and MSA-IL-2 or MSA-oIL-2. **P < 0.01 (ANOVA). **e**, Survival of mice treated with pmel T cells and MSA-IL-2 or MSA-oIL-2 for the indicated times. NS, not significant; **P < 0.01 (log-rank test). **f**, Quantification of tumour-infiltrating o2R or o9R pmel T cells five days after ACT in mice treated with MSA-oIL-2 (*n* = 5 mice per group). *P < 0.05 (unpaired *t*-test). **g**, In vitro growth of nRFP^+^ B16-F10 tumour cells cocultured with pmel T cells (2:1 effector: target (E:T) ratio) pretreated with MSA-oIL-2 (5 μM). **P < 0.01 (ANOVA). **h**, opt-SNE clustering of o2R and o9R pmel T cells treated with MSA-oIL-2 (5 μM) for 48 h in vitro (left), with separate plots by group showing differentially abundant clusters (middle), and an annotated volcano plot (right). **i**, Heat maps of manually curated genes associated with T cell stemness and dysfunction (left) and activation and effector function (right), and differentially expressed between o2R and o9R pmel T cells (from Fig. 3h) treated with MSA-oIL-2 (5 μM). MSA-IL-2-treated groups (50 nM) are also shown. **j**, Plot of the normalized enrichment score (NES) (left) of transcription factor gene sets comparing o2R and o9R pmel T cells treated with MSA-oIL-2 (from Fig. 3h). Significant enrichment in red (adjusted *P* < 0.05, two-sided Fisher’s test with hypergeometric formula). Right, ratio of *Jun* to *Fos* expression. **P < 0.01 (unpaired *t*-test). Data are mean ± s.d. with *n* = 3 biological replicates, unless stated otherwise. Source data

**Extended Data Fig. 4:**
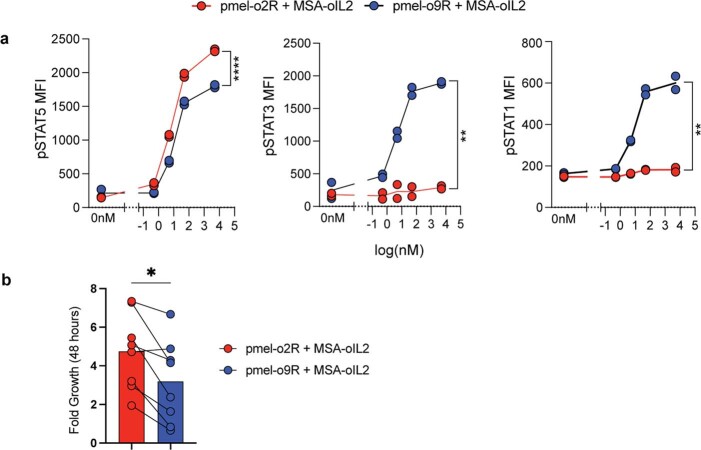
Signalling and proliferation of o2R and o9R pmel T cells. **a**, STAT1, STAT3 and STAT5 phosphorylation in o2R versus o9R pmel T cells after 30’ stimulation with MSA-oIL2 (5μM). Shown are MFI of individual biological duplicates, gated on YFP+ cells. **, p < 0.01; ****, p < 0.0001 (ANOVA). **b**, Shown are seven replicate experiments measuring in vitro proliferation (measured by fold growth) of o2R or o9R pmel T cells over 48 h in culture with MSA-oIL2, (each data point represents mean ± SD, n = 3). *, p < 0.05 (ratio paired t-test, two-sided).

Given the unique STAT signalling profile, its lesser-known status among the γ_c_ cytokine receptor family and the acquisition of features of stem cell memory T (T_SCM_) cells, we chose to study the effect of o9R signalling in vivo using mouse models of ACT for solid tumours. We first used T cells from transgenic pmel mice, which express an endogenous T cell receptor (TCR) specific for gp100, a melanocytic antigen that is overexpressed in B16 melanoma^38^. We modified the protocol to be more stringent and omitted lymphodepleting radiotherapy, a conditioning regimen that potentiates ACT by inducing the homeostatic proliferation of adoptively transferred T cells through γ_c_ cytokine signalling^39^ (Fig. 2a). The STAT signalling profile and proliferation of o2R and o9R pmel T cells mirrored those of o2R and o9R T cells from C57BL/6 mice (Extended Data Fig. 4a,b).

**Extended Data Fig. 5:**
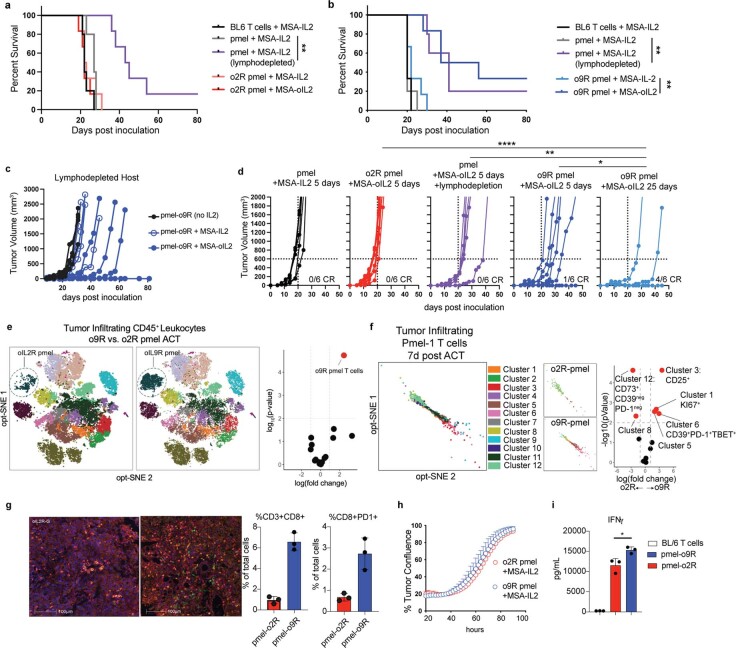
Anti-tumour efficacy, tumour infiltration, phenotype and function of o2R and o9R pmel T cells. **a**,**b**, Survival of B16-F10 tumour-bearing mice treated with (**a**) o2R or (**b**) o9R pmel T cells and MSA-IL2 or MSA-oIL2. BL/6 T cells treated with MSA-IL2 were used as an off-target T cell control. Untransduced pmel T cells plus mIL-2 in tumour-bearing lymphodepleted and non-lymphodepleted mice served as controls. **c**, Effect of o9R pmel T cells in a lymphodepleted host. Tumour growth (mean ±SEM, left panel) of B16-F10 tumours in lymphodepleted C57BL/6 mice treated with o9R pmel T cells and MSA-IL2, MSA-oIL2, or no IL2. **d**, Individual B16-F10 tumour growth curves related to survival curves shown in Fig. 2e. All mice were non-lymphodepleted except one group (in purple) which received total body irradiation (5Gy). CR, complete regression. *, p < 0.05; **p < 0.01; ****, p < 0.0001 (ANOVA). **e**, opt-SNE clustering of CD45^+^ tumour-infiltrating leukocytes seven days after adoptive transfer of o2R (left) and o9R (middle) pmel T cells (n = 4 mice/group; mice were treated without lymphodepletion and treated MSA-oIL2 2.5x10^4^ units/day for five days starting with ACT). Volcano plot of differentially abundant clusters in tumours from mice treated with o9R versus o2R pmel T cells (right panel). **f**, opt-SNE clustering of the subset of tumour infiltrating o9R and o2R pmel T cells in non-lymphodepleted hosts treated with MSA-oIL2 (n = 4 mice/group, left panel), with separate plots for each treatment group illustrating only differentially abundant clusters (middle panel), and a volcano plot of differentially abundant clusters annotated with distinguishing features (right panel). **g**, Tumour-infiltration of CD3^+^CD8^+^ T cells and CD8^+^PD1^+^ T cells in non-lymphodepleted hosts treated with either o2R and MSA-oIL2 or o9R pmel T cells and MSA-oIL2 by multiplex IHC (red = CD3, orange = CD8, yellow = PD1, green = CD4, teal = FOXP3). Images are representative of tumours from n = 3 mice/group and of one independent experiment (conclusions verified in three independent experiments by flow and mass cytometry). Quantifications (mean ± SD) shown to the right (n = 3 biological replicates/group). **h**, In vitro growth (mean ± SD, n = 3 biological replicates/group) of nRFP^+^ B16-F10 tumour cells cocultured with o2R or o9R pmel T cells (2:1 E:T ratio) pretreated with MSA-IL2 (50nM). **i**, IFNγ secretion by oIL-2R and oIL-9R pmel T cells cocultured with B16-F10 melanoma in vitro for 24 h. T cells were pretreated with MSA-oIL2 (5 µM) for 48 h in vitro prior to coculture (mean ± SD, n = 3 biological replicates/group). *, p < 0.05; unpaired t-test, two-sided. Source data

Although both o2R and o9R pmel T cells expanded in the absence of lymphodepletion (Fig. 2b), we observed more consistent anti-tumour effects and prolonged survival in this model with o9R pmel T cells treated with MSA-oIL-2 (Fig. 2c,d and Extended Data Fig. 5a,b), achieving anti-tumour effects comparable to pmel T cells in lymphodepleted mice. And although T cells engineered to secrete native cytokines can also obviate the need for lymphodepletion^40,41^, native cytokines (unlike orthogonal cytokine signalling) induce signalling in both adoptively transferred and endogenous cells. The superior anti-tumour efficacy of o9R pmel T cells was also observed in lymphodepleted mice that were treated with MSA-oIL-2 (Extended Data Fig. 5c).

As MSA-oIL-2 clone 3A10 is highly orthogonal and does not affect endogenous cells, we hypothesized that mice would tolerate a prolonged dosing regimen of 25 days, whereas the toxic effects of MSA-IL-2 are observed after 5 days^9^. We did not observe clinical toxicity with the prolonged dosing regimen, which resulted in superior tumour control and survival with complete regressions in four of six B16 tumour-bearing mice (Fig. 2e and Extended Data Fig. 5d), exceeding the results in lymphodepleted mice treated with pmel ACT and five days of MSA-IL-2.

These results, despite the weaker proliferative effect of o9R signalling, suggested that factors beyond selective expansion underlie its enhanced anti-tumour effects. We observed greater tumour infiltration by o9R pmel T cells compared to o2R pmel T cells five days after ACT in mice that were treated with MSA-oIL-2 (Fig. 2f and Extended Data Fig. 5g). Among 18 tumour-infiltrating CD45^+^ leukocyte population clusters, the dominant distinguishing feature was the presence of o9R versus o2R pmel T cells (8.9% versus 1.6% of tumour infiltrating CD45^+^ cells; Extended Data Fig. 5e). Compared to o2R tumour-infiltrating pmel T cells, o9R pmel T cells were enriched for clusters that are associated with T cell activation, including a cluster that co-expressed CD39, PD-1 and TBET (Extended Data Fig. 5f). In addition to improved tumour infiltration, o9R pmel T cells showed a higher in vitro cytolytic capacity and increased production of IFNγ compared to their o2R counterparts (Fig. 2g and Extended Data Fig. 5h,i).

**Extended Data Fig. 6:**
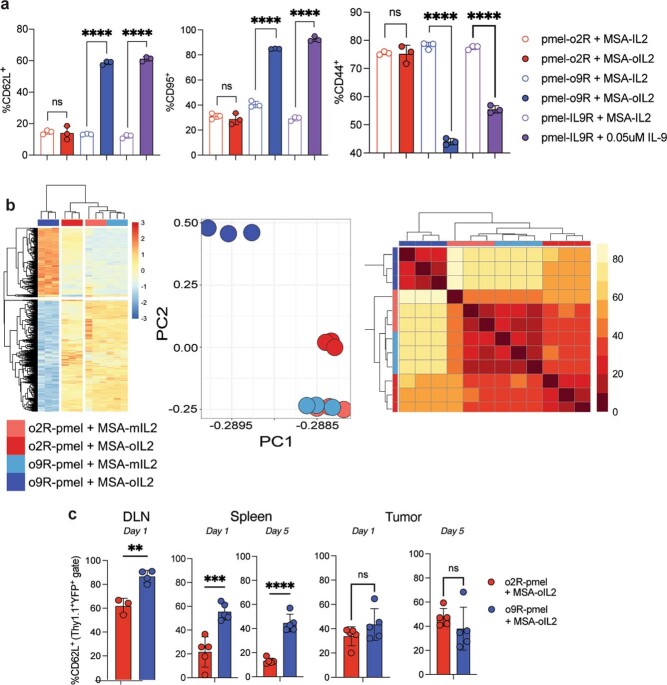
o9R signalling in vitro and in vivo drives a T_SCM_ phenotype in pmel T cells. **a**, Surface expression of CD62L, Fas (CD95) and CD44 as percentage of CD8^+^Thy1.1^+^ sorted o9R pmel T cells, o2R pmel T cells, or pmel T cells transduced with wild-type IL-9R (pmel-IL-9R) and treated with MSA-IL2 (0.05 μM), MSA-oIL2 (5 μM), or IL-9 (.05 μM) for 48 h in vitro. Shown are mean ±SD, n = 3 biological replicates/group. ns, not significant; ****, p < 0.0001; unpaired t-tests, two-sided. **b**, unsupervised transcriptomic analysis (RNA-seq) of sorted pmel-o2R (n = 3) and pmel-o9R (n = 3) T cells 48 h after exposure to MSA-oIL2 or MSA-IL2 in vitro. Heat map of genes differentially expressed between o2R and o9R pmel T cells treated with MSA-oIL2 (5 μM)(left panel). MSA-IL2 treated groups (50nM) also shown. On principal component analysis (PC1 v PC2, top right panel), samples separate by treatment group. Samples cluster by treatment group when arranged by sample-sample distances in a heat map (bottom right panel), with o9R pmel T cells treated with oIL-2 most distinct among the four groups. **c**, Frequency of CD62L^+^ o2R or o9R pmel T cells (Thy1.1^+^YFP^+^) from tumour draining lymph nodes (DLN), spleen or tumours of mice treated with MSA-oIL2 (without prior lymphodepletion). Tissues were collected one or five days after adoptive transfer. Shown are mean ±SD with individual data points (n = 3-5 mice per group). **, p < 0.01; ***, p < 0.001; ****, <0.0001; unpaired t-test, two-sided.

We then investigated the biological program that is responsible for the improved infiltration, effector function and in vivo efficacy of o9R pmel T cells. In vitro exposure to MSA-oIL-2 resulted in markedly divergent phenotypes by high-dimensional mass cytometry; 8 of 14 clusters were differentially abundant between o2R and o9R pmel T cells (Fig. 2h). o9R pmel T cells acquired markers of a T_SCM_ phenotype (Sca-1, CD127, Fas and CD62L), mirroring the phenotypic effects of wild-type IL-9R signalling (Fig. 2h and Extended Data Fig. 6a). These T_SCM_ features were among the global bidirectional transcriptomic changes observed by RNA sequencing (RNA-seq) in a bulk population of pmel T cells (Extended Data Fig. 6b). However, the changes induced by o9R extend beyond the acquisition of a T_SCM_ phenotype; we also observed the simultaneous enrichment of genes classically associated with T cell activation (*Pdcd1*, *Icos*, *Entpd1*, *Lag3* and *Havcr2*) and effector function (*Ifng*, *Gzma* and *Prf1*), which are traditionally excluded from the T_SCM_ phenotype (Fig. 2i). This may represent a hybrid phenotype or the simultaneous presence of heterogeneous subpopulations unique to o9R or native IL-9R signalling in T cells. The induction of granzyme A by IL-9R activation has been reported to be dependent on the concomitant activation of STAT1 and STAT3, a characteristic of o9R signalling that is distinct from o2R, o4R, o7R and o21R signalling^14^.

A transcriptomic analysis of transcription factor pathway enrichment revealed a significant enrichment of genes associated with the AP-1 transcription factor JUN, in addition to the expected transcription factors STAT1 and STAT3 (Fig. 2j). This was accompanied by an increase in the ratio of *Jun* to *Fos* expression, a feature of tumour-specific T cells that are resistant to tumour-induced exhaustion^42^. In parallel, o9R signalling downregulated genes that are associated with T cell dysfunction, including *Nr4a1* and *Tox* (refs. ^43–46^; Fig. 2i).

To examine the in vivo activity of o9R signalling, we examined the expression of CD62L in adoptively transferred pmel T cells. CD62L expression was higher in o9R than in o2R pmel T cells in the draining lymph nodes and spleens of tumour-bearing mice treated with MSA-oIL-2 (Extended Data Fig. 6c). No difference was observed intratumorally, in which antigen-specific T cell activation is likely to predominate.

**Fig. 3:**
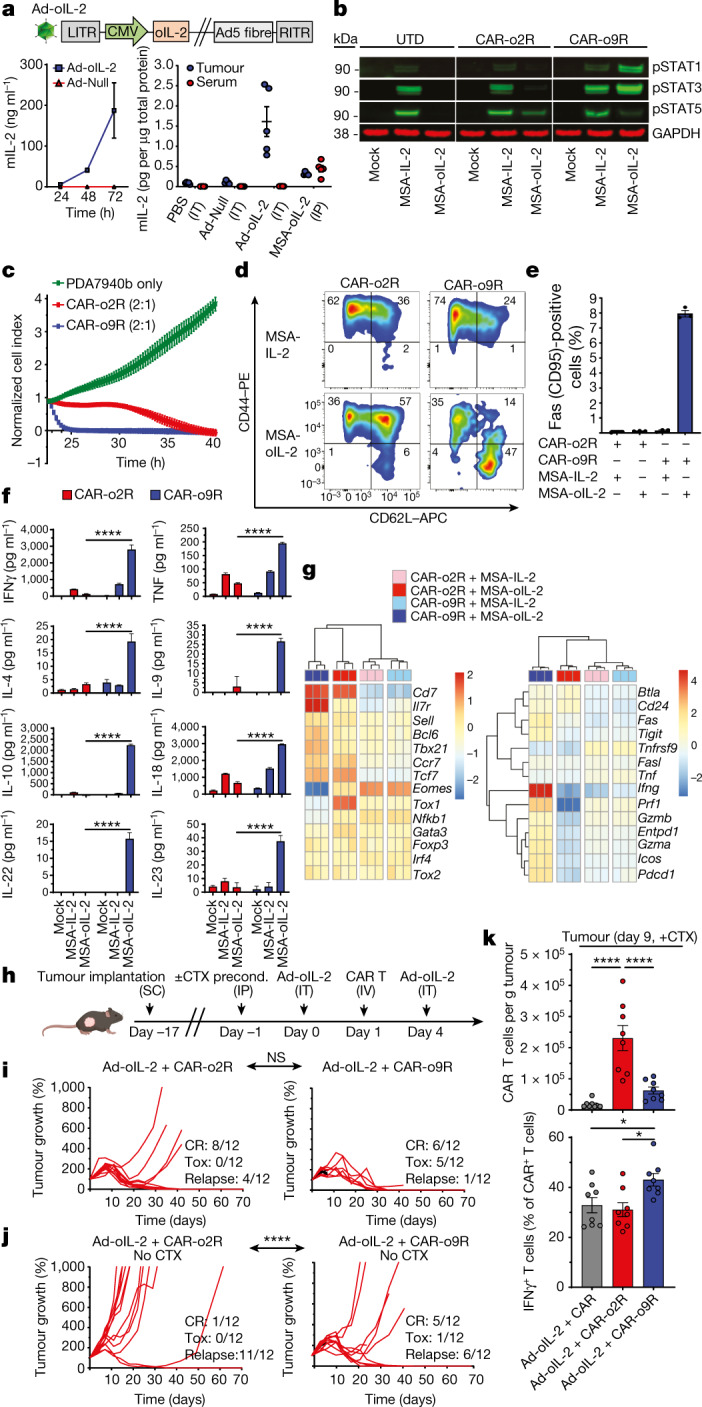
Tumour-restricted o9R signalling improves the potency of CAR T cells. **a**, Top, schematic of an adenoviral vector encoding oIL-2 (Ad-oIL-2) under the cytomegalovirus (CMV) promoter. LITR, left inverted terminal repeat; RITR, right inverted terminal repeat. Bottom left, in vitro expression of oIL-2 through Ad-oIL-2 in cell culture supernatants. mIL-2, mouse IL-2. Bottom right, quantification of oIL-2 in tumour homogenates and sera 72 h after intratumoral (IT) injection of 10^9^ viral particles (VP) of Ad-oIL-2, or daily intraperitoneal (IP) injection of 2.5 × 10^4^ units MSA-oIL-2 (*n* = 5 mice per group). **b**, Representative western blot analysis of pSTAT1, pSTAT3 and pSTAT5 expression in T cells 30 min after stimulation with MSA-IL-2 (100 nM) or MSA-oIL-2 (5 μM). For gel source data, see Supplementary Fig. 1. **c**, In vitro T cell killing of mesothelin-positive PDA7940b (2:1 E:T ratio) pre-incubated with MSA-oIL-2 (5 μM) (mean ± s.d., *n* = 4 per group). **d**–**f**, Representative surface expression of CD44 and CD62L (**d**), expression of Fas (CD95) (**e**), and secreted cytokines (**f**) in CAR-o2R or CAR-o9R T cell cultures after four days of stimulation with MSA-IL-2 (100 nM) or MSA-oIL-2 (5 μM). ****P < 0.0001 (ANOVA). **g**, Heat maps of genes associated with T cell stemness and dysfunction (left) and activation and effector function (right), and differentially expressed between o2R and o9R CAR T cells treated with MSA-oIL-2. **h**, Schematic of the syngeneic ACT model using PDA7940b tumours (created with Biorender.com). Ad-oIL-2 dose, 10^9^ VP. CAR T cell dose, 5 × 10^6^ cells. Cyclophosphamide (CTX) dose, 120 mg kg^−1^. IV, intravenous; precond., preconditioning; SC, subcutaneous. **i**,**j**, Individual growth curves of PDA7940b tumours (*n* = 12 mice per group), with (**i**) and without (**j**) conditioning CTX. Black lines indicate deaths due to ICANS. *n* = 12 mice per group. CR, complete response; Tox, deaths due to neurotoxicity. NS, not significant; ****P < 0.0001 (ANOVA). **k**, Quantification of tumour-infiltrating CAR T cells (top) and frequency of IFNγ-positive tumour-infiltrating CAR T cells (bottom) on day 9 in mice treated with CTX. *P < 0.05, ****P < 0.0001 (ANOVA). Data are mean ± s.e.m. with *n* = 3 biological replicates, unless stated otherwise. Source data

To investigate o9R signalling in the context of chimeric antigen receptor (CAR)-based ACT, we used an immunotherapy-resistant model of pancreatic cancer expressing mesothelin. As the source of oIL-2 for CAR T cell studies, we chose vectored intratumoral delivery (Fig. 3a) to ensure high concentrations of oIL-2 (clone 3A10) in the tumour and to evaluate the effect of o9R signalling on T cell dysfunction in the tumour microenvironment. This model provided a contrast to pmel studies, in which o9R signalling was most active in peripheral tissues.

**Extended Data Fig. 7:**
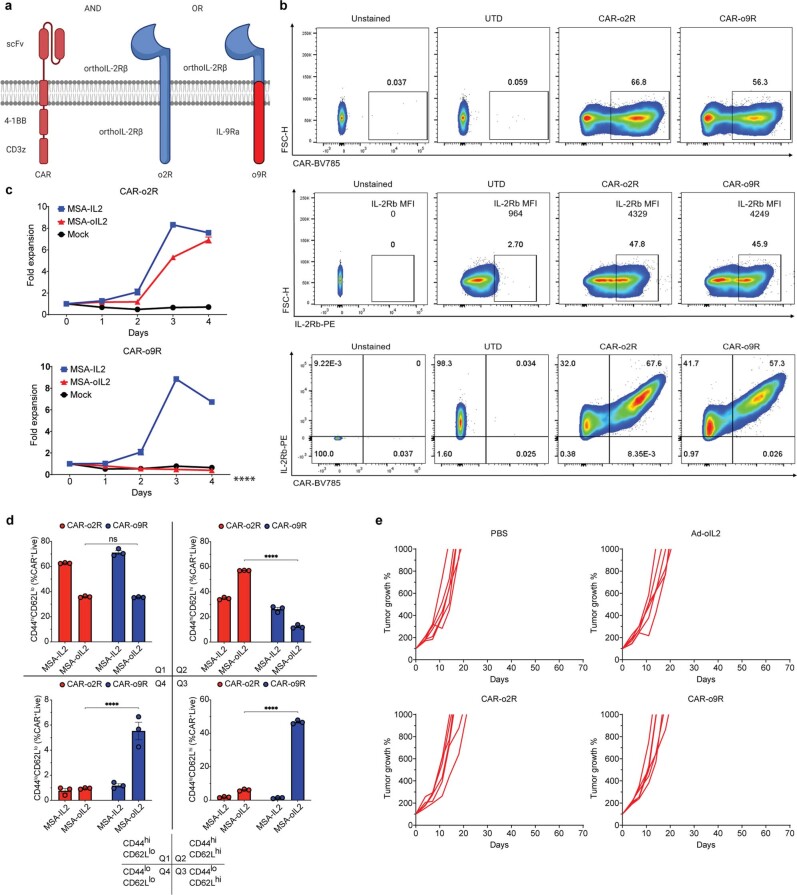
o2R and o9R signalling in T cells engineered with an anti-mesothelin CAR. **a**, Schematic of primary mouse CD3^+^ T cells expressing anti-mesothelin CAR and o2R (CAR-o2R) or o9R (CAR-o9R). **b**, Representative mesothelin CAR expression (top panel), IL-2Rβ expression (middle panel) and CAR/IL-2Rβ co-expression (bottom panel) in untransduced (UTD) or retrovirally transduced CAR-o2R and CAR-o9R T cells as determined by flow cytometry. Middle panel inset, mean fluorescence intensity (MFI) of IL-2Rβ in untransduced and transduced T cell. **c**,**d**, Expansion and phenotype of o2R and o9R CAR T cells. **c**, In vitro CAR T cell expansion. CAR-o2R and CAR-o9R cells were incubated in the presence of MSA-IL2 (100nM) or MSA-oIL2 (5 μM). An aliquot of cells was removed from the plate and stained with Calcein AM viability dye and counted on the Celigo Image Cytometer daily. Mean ±SEM, n = 3 replicate wells/group. **d**, CD44 and CD62L co-expression on CAR T cells. Full data of representative flow plots in Fig 3d. CAR-o2R and CAR-o9R cells were incubated for four days in the presence of MSA-IL2 (100nM) or MSA-oIL2 (5 μM). CD44 and CD62L surface co-expression was determined by flow cytometry on live CAR+ cells. Mean ±SEM, n = 3/group. ns, not significant. ****P < 0.0001 (ANOVA). **e**, Individual growth curves of PDA7940b tumours by treatment group; control groups corresponding to Fig. 3i,j Source data

We transduced primary mouse T cells with retroviruses that encode a second-generation anti-mouse mesothelin CAR together with orthogonal receptors to generate CAR-o2R and CAR-o9R T cells (Extended Data Fig. 7a,b). Stimulation of CAR-o9R and CAR-o2R T cells with MSA-oIL-2 reproduced the STAT phosphorylation and proliferation profiles of o9R and o2R signalling observed in Figs. 1 and 2 (Fig. 3b and Extended Data Fig. 7c). Despite their proliferative disadvantage, CAR-o9R cells showed superior anti-tumour efficacy in vitro against the mesothelin-positive pancreatic ductal adenocarcinoma (PDA) cell line PDA7940b (Fig. 3c).

Similar to the pmel model, CAR-o9R cells were characterized by a T_SCM_ phenotype after stimulation with MSA-oIL-2 (Fig. 3d,e and Extended Data Fig. 7d). CAR-o9R T cells also secrete higher levels of IFNγ, TNF, IL-4, IL-9, IL-10, IL-18, IL-22 and IL-23 than CAR-o2R cells in response to oIL-2 (Fig. 3f), mirroring the complex transcriptomic signature of o9R pmel T cells. This was validated at the transcriptomic level; genes that are associated with effector functions (*Ifng*, *Prf1*, *Gzmb* and *Gzma*) and T cell activation (*Pdcd1*, *Icos* and *Entpd1*) were upregulated in CAR-o9R cells, along with a shift toward stemness (upregulation of *Il7r*, *Sell*, and *Bcl6* and downregulation of *Eomes* and *Tox1*; Fig. 3g).

In a recalcitrant model of PDA using established subcutaneous PD7940b tumours, treatment either with an adenoviral vector encoding oIL-2 (Ad-oIL-2) or with CAR-o2R or CAR-o9R was ineffective in controlling rapidly growing tumours (Fig. 3h and Extended Data Fig. 7e). However, combination therapy with Ad-oIL-2 plus CAR-o2R or Ad-oIL-2 plus CAR-o9R resulted in complete regressions in 8 of 12 mice (67%) and 6 of 12 mice (50%), respectively (Fig. 3i). Early toxicity was observed in the Ad-oIL-2 plus CAR-o9R group, as 40% of mice (5 of 12) died by day 10, but—notably—fewer tumour relapses were seen in surviving mice in the Ad-oIL-2 plus CAR-o9R group (1 of 7) versus the Ad-oIL-2 plus CAR-o2R group (4 of 12).

**Extended Data Fig. 8:**
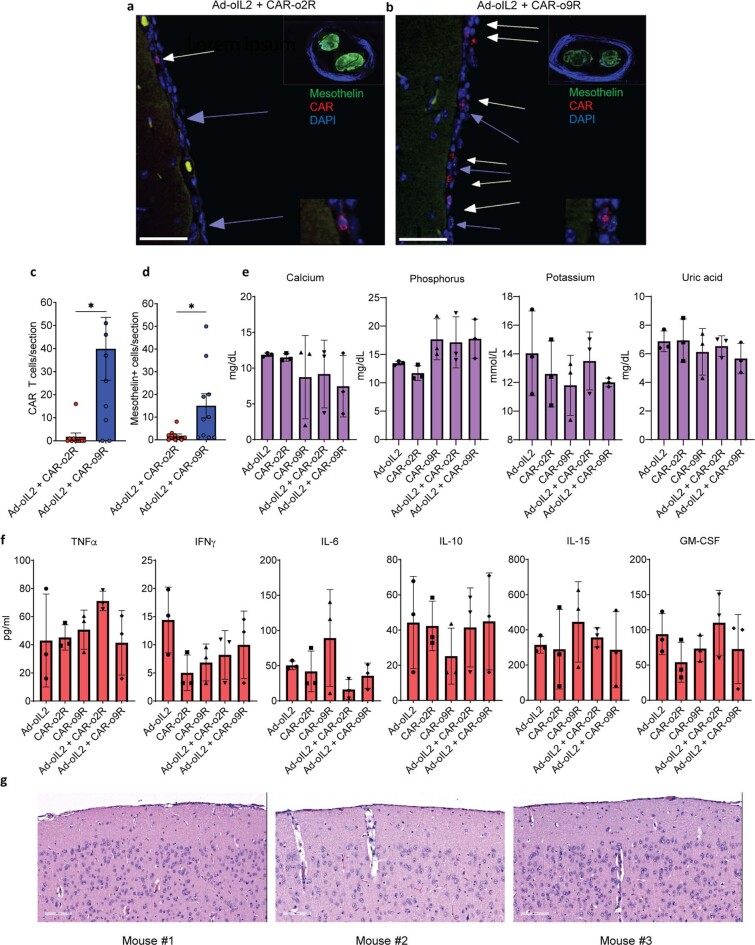
RNA ISH and serum markers of toxicity on day 11. **a**,**b**, Representative images of brain and meningeal sections stained with fluorescent probes specific for mouse CAR (red, Cy3), mouse mesothelin (green, FITC) and counterstained with DAPI (blue). CAR-positive cells (white arrows) and mesothelin-positive meningeal cells (purple arrows) indicated in the pia-arachnoid layer of the meninges. Scale bars, 50 μm. **c**,**d**, Semi-quantification of CAR T cells and mesothelin-positive cells in stained brain sections. Mean ±SEM, two sections/mouse, three mice/group. *P < 0.05 (two-sided *t* test with Welch’s correction). **e**, Serum levels of calcium, phosphorus, potassium, uric acid, and **f**, cytokine release syndrome (CRS)-associated cytokines on day 11 of treatment. Mean ± SEM, *n* = 3 mice/group. **g**, Representative photomicrographs from 3 mice (160 days post-treatment) cured of PDA7940b tumours showing histologically normal leptomeninges, without immune/inflammatory cell infiltrates, covering the temporoparietal region of the cerebral hemispheres. H&E, scale bar: 100 μm.

The toxicity of Ad-oIL-2 plus CAR-o9R in lymphodepleted mice was characterized by clinical signs (tremor, delirium and seizures) of immune-effector-cell-associated neurotoxicity syndrome (ICANS). An RNA in situ hybridization (RNA ISH) analysis of mice with ICANS revealed that CAR T cells infiltrated into the meningeal layers of the brain, where mesothelin-expressing meningeal cells were also detected (Extended Data Fig. 8a,b). Significantly more meningeal-infiltrating CAR T cells were observed in the Ad-oIL-2 plus CAR-o9R group versus the Ad-oIL-2 plus CAR-o2R group, suggesting an association between CAR-driven on-target off-tumour activity and the observed ICANS (Extended Data Fig. 8c). This was accompanied by higher expression of mesothelin, which can occur in the context of an inflammatory stimulus (Extended Data Fig. 8d). Serum analyses did not show evidence of cytokine release syndrome or tumour lysis syndrome (Extended Data Fig. 8e,f). Histological examination of the brains of three long-term surviving mice that were treated with Ad-oIL-2 plus CAR-o9R was unremarkable, including normal leptomeninges without inflammatory cells (Extended Data Fig. 8g). On the basis of these findings and the absence of similar toxicities in the pmel model, the observed ICANS appears to be specific to the CAR specificity and not inherent to o9R T cells. Still, clinical translation of o9R must consider potential added on-target off-tumour toxicity, and may require engineering strategies (for example, on/off systems and synthetic circuits) to maximize patient safety.

**Extended Data Fig. 9:**
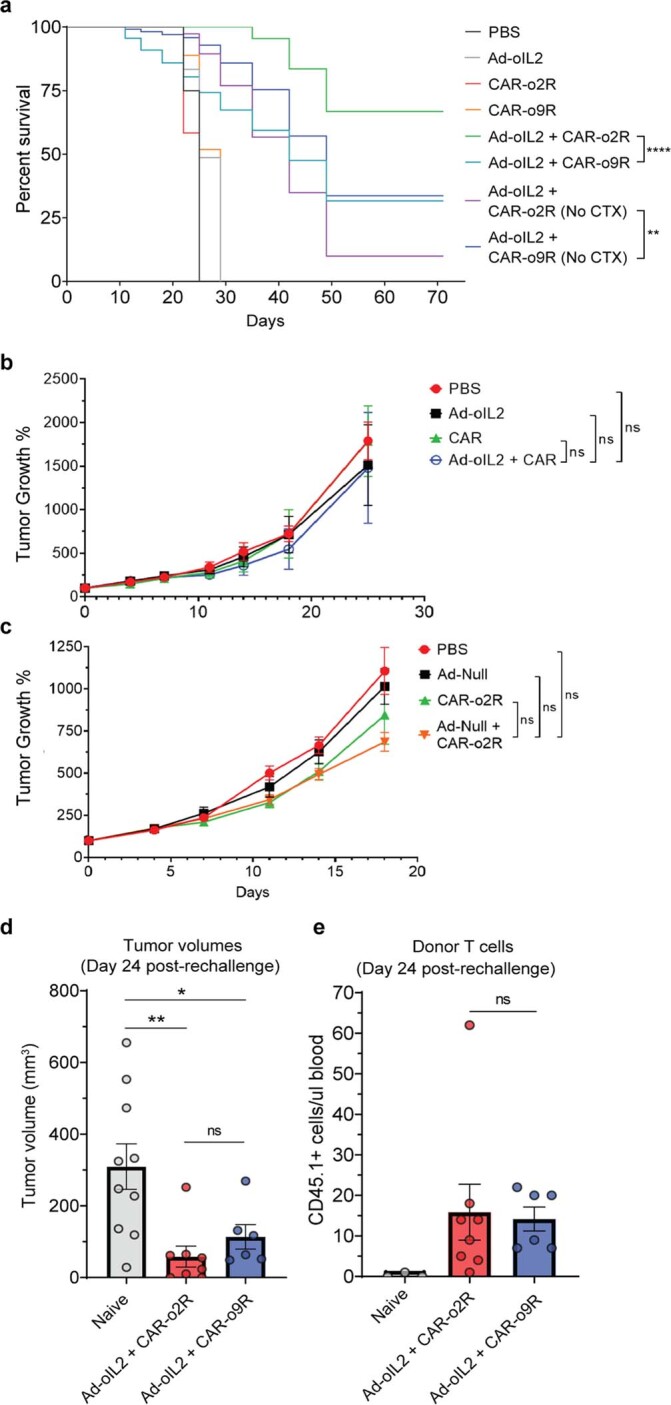
Anti-tumour efficacy of mesothelin-specific o2R and o9R CAR T cells combined with intratumoral delivery of Ad-oIL2. **a**, Survival differences between mice treated with CAR-o2R + Ad-oIL2 and CAR-o9R T cells + Ad-oIL2 with or without conditioning chemotherapy. Kaplan-Meier survival curves of treatment groups in Fig. 3i–k. **P < 0.01, ****P < 0.0001 (Log rank Mantel-Cox test). **b**,**c**, Anti-tumour efficacy of Ad-oIL2 with control CAR T cells or Ad-Null and CAR-o2R T cells. **b**, Anti-tumour efficacy in mice treated with Ad-oIL2 and CAR T cells (without orthogonal cytokine receptor). Established subcutaneous PDA7940b tumours were treated with Ad-oIL2 on days 0 and 4 (1x10^9^ VP/tumour) and with CAR T cells on day 0 (5x10^6^). Mean ± SEM, n = 5 mice/group. ns, not significant (ANOVA). **c**, Anti-tumour efficacy in mice treated with Ad-Null (no transgene) and CAR-o2R T cells. Established subcutaneous PDA7940b tumours were treated with Ad-Null on days 0 and 4 (1x10^9^ VP/tumour) and with CAR-o2R T cells on day 0 (5x10^6^). Mean ± SEM, n = 5 mice/group. ns, not significant (ANOVA). **d**, Tumour volumes (mean ±SEM) of cured mice (from Fig. 3j) rechallenged with PDA7940b compared to age-matched naïve mice (n = 10, 8 and 6 mice for naïve, Ad-oIL2 + CAR-o2R and Ad-oIL2 + CAR-o9R groups, respectively). *P < 0.05; **P < 0.01; ns, not significant (ANOVA). **e**, Quantification of CD45.1+ lymphocytes in peripheral blood of rechallenged mice (mean ±SEM, n = 10, 8 and 6 mice for naïve, Ad-oIL2 + CAR-o2R and Ad-oIL2 + CAR-o9R groups, respectively). ns, not significant (ANOVA). Source data

In the absence of conditioning chemotherapy, the superiority of the Ad-oIL-2 (3A10) plus CAR-o9R regime over Ad-oIL-2 (3A10) plus CAR-o2R was more evident, as suggested by complete regression rates of 42% (5 of 12) and 8.3% (1 of 12), respectively (Fig. 3j), as well as by prolonged survival (Extended Data Fig. 9a), as only 1 of 12 mice in the Ad-oIL-2 plus CAR-o9R group exhibited toxicity in the absence of conditioning. Efficacy was dependent on both orthogonal receptor expression in T cells and oIL-2 expression through Ad-oIL-2 (Extended Data Fig. 9b,c).

We examined the effect of Ad-oIL-2 on the quantity and quality of tumour-infiltrating CAR-o9R or CAR-o2R T cells. In contrast to the pmel model, fewer CAR-o9R than CAR-o2R T cells were observed in the tumour eight days after ACT (Fig. 3k). Comparisons between the models must be made cautiously, given the differences between CAR and TCR signalling and between tumour-restricted and systemic cytokine distribution. Nonetheless, the higher frequency of intratumoral CAR-o9R cells expressing IFNγ (Fig. 3k) and the superior direct in vitro cytotoxicity of CAR-o9R cells (Fig. 3c) indicate that superior intratumoral potency—rather than tumour infiltration or proliferation—drives the anti-tumour efficacy of CAR-o9R T cells in the context of tumour-restricted orthogonal cytokine signalling. By contrast, systemic administration of oIL-2 in the pmel model resulted in peripheral o9R signalling (Extended Data Fig. 6c), which may explain the enhanced trafficking of o9R pmel T cells. Peripheral o9R signalling may also facilitate interactions between adoptively transferred T cells and endogenous cells (for example, antigen-presenting cells) that help to sustain anti-tumour effects.

**Fig. 4:**
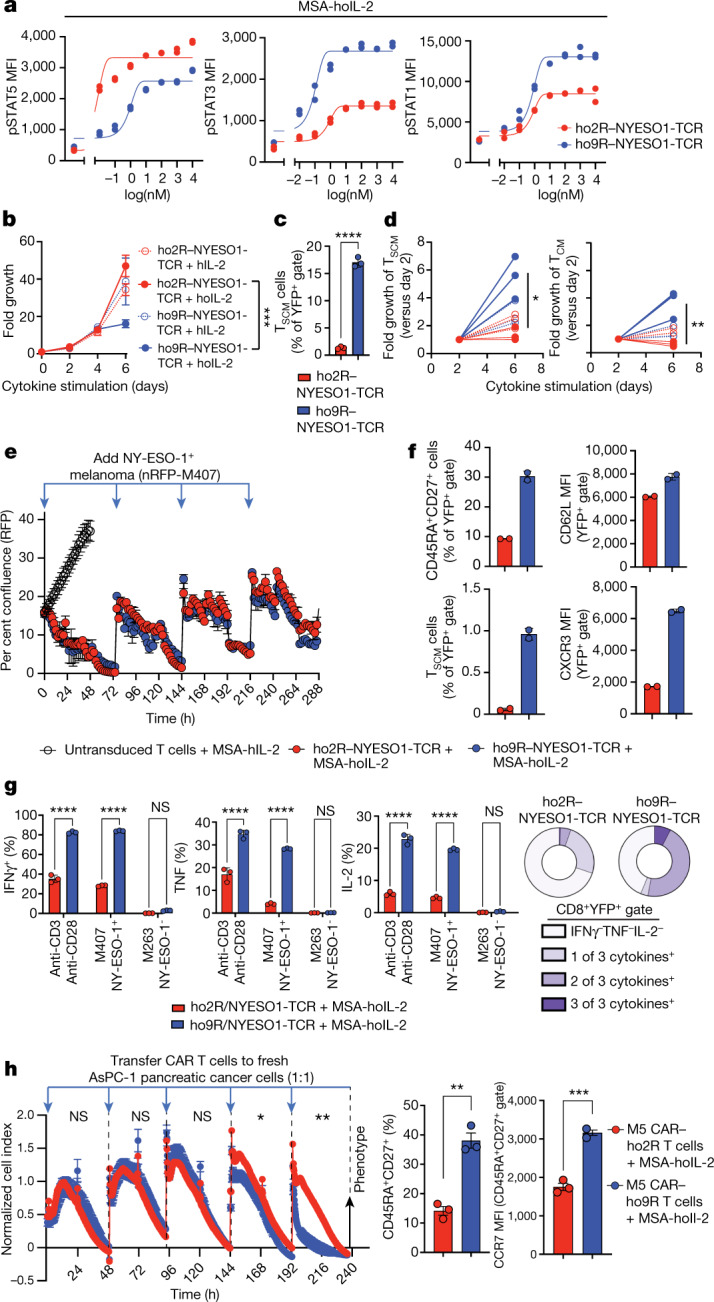
Human chimeric orthogonal IL-2Rβ-ECD–IL-9R-ICD drives stemness and a superior effector capacity in TCR- and CAR-engineered T cells. **a**, pSTAT signalling in human NY-ESO-1 TCR T cells co-expressing ho2R or ho9R and stimulated with MSA-hoIL-2 for 20 min. **b**, Fold expansion of T cells treated with MSA-hoIL-2 (1 μM) or MSA-hIL-2 (0.1 μM) (mean ± s.d.; *n* = 3 per group). ***P < 0.001 (unpaired *t*-test at day 6). **c**, Percentage of CD45RA^+^CD27^+^CD95^+^CCR7^+^ T_SCM_ cells after six days in culture with MSA-hoIL-2 (1 μM)(mean ± s.d.; *n* = 3 per group). ****P < 0.0001 (unpaired *t*-test). **d**, Fold expansion of T_SCM_ and T_CM_ cells with MSA-hoIL-2 (1 μM) or MSA-hIL-2 (0.1 μM), relative to day 2. *P < 0.05, **P < 0.01 (unpaired *t*-test); *n* = 3 per group. **e**, T cells were cocultured at a 1:1 E:T ratio with the HLA*0201^+^ NY-ESO-1^+^ melanoma cell line (nRFP-M407) and MSA-hoIL-2 (1 μM). Tumour cells (10^5^) were reintroduced every 72 h (blue arrows) in the presence of MSA-hoIL-2 (1 μM). Shown is the percentage tumour confluence (mean ± s.d.; *n* = 3 per group). **f**, Percentage of CD45RA^+^CD27^+^ and T_SCM_ cells, alongside CD62L and CXCR3 MFI (YFP^+^ gate) after the fourth tumour challenge (from **e**). **g**, T cells after the fourth tumour challenge (from **e**) were restimulated with anti-CD3 or anti-CD28 antibodies, M407 (HLA*0201^+^NY-ESO-1^+^) or M263 (HLA*0201^−^NY-ESO-1^−^). IFNγ, TNF and IL-2 were quantified among CD8^+^YFP^+^ T cells by intracellular cytokine staining (ICS) (mean ± s.d.; *n* = 3 per group). NS, not significant; ****P < 0.0001 (two-way ANOVA). Donut charts indicate the proportion of CD8^+^YFP^+^ T cells in each group expressing 0/3, 1/3, 2/3 or 3/3 cytokines. **h**, Sorted T cells co-expressing either ho2R or ho9R and anti-mesothelin M5 CAR were cocultured at a 1:1 E:T ratio with AsPC-1 PDA cells in the presence of MSA-hoIL-2 (1 μM) every 48 h. Left, tumour viability measured as normalized cell index (mean ± s.e.m.; *n* = 3 per group). Right, after the last tumour challenge, T cell surface markers were characterized. *P < 0.05, **P < 0.01, ***P < 0.001; NS, not significant (two-way ANOVA for tumour cell killing; unpaired *t*-test for phenotypic analysis). Source data

**Extended Data Fig. 10:**
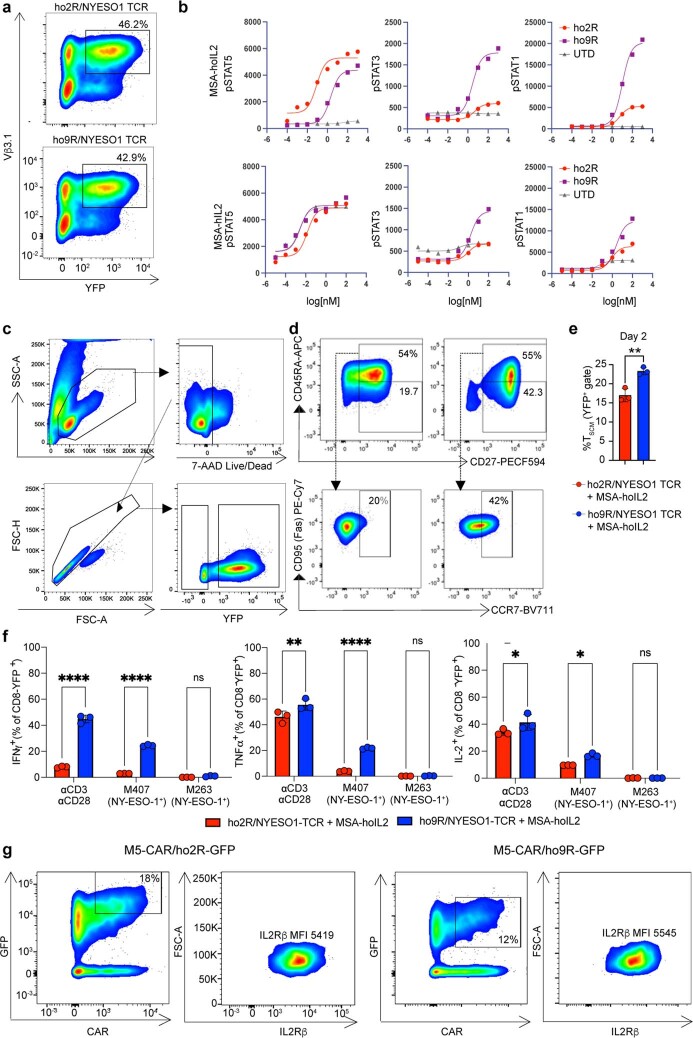
Human chimeric orthogonal IL-2Rβ-ECD–IL-9R-ICD drives a stem-like phenotype and polyfunctionality in T cells even after repetitive antigen-specific tumour challenge. **a**, Flow plots of normal healthy donor T cells co-transduced with either ho2R and NYESO1-TCR (left) or ho9R and NYESO1-TCR (right), as detected by anti-human Vβ13.1 antibody that recognizes the β chain of the NYESO1-TCR clone 1G4 and YFP (internal marker of the o2R or o9R vectors). Co-transduction efficiencies are shown. **b**, pSTAT signalling dose response of untransduced or ho2R (red) or ho9R (purple) or untransduced (UTD; grey) expressing activated human T cells (donor 651) stimulated with MSA-hoIL2 (top panel) or MSA-hIL2 (bottom panel) for 20’. Data are shown as mean fluorescence ± SEM, n = 2. **c**, Gating strategy for immunophenotyping of YFP^+^ population of ho2R–NYESO1-TCR and ho9R–NYESO1-TCR T cells shown in Fig. 4c. **d**, Immunophenotype of ho2R–NYESO1-TCR and ho9R–NYESO1-TCR engineered human T cells after six days in culture with MSA-hoIL2 (1 μM). Shown are representative plots of CD45RA and CD27 expression gated on YFP^+^ T cells (top row), and CD95 and CCR7 expression gated on the CD45RA^+^CD27^+^ population (indicated by arrow and dotted line) corresponding to bar plot in Fig. 4c. **e**, Immunophenotype of ho2R–NYESO1 TCR and ho9R–NYESO1 TCR engineered human T cells after two days in culture with MSA-hoIL2 (1 μM). Shown is bar plot quantification of T_SCM_ cells as a percentage of the YFP^+^ population (mean ±SD, n = 3 biological replicates/group). **, p < 0.01 (unpaired t-test, two-sided). **f**, T cells from Fig. 4e were collected and restimulated with either αCD3/αCD28 dynabeads, melanoma cell line M407 (HLA*0201^+^NY-ESO-1^+^) or M263 (HLA*0201^+^NY-ESO-1^−^). IFNγ, TNF and IL-2 were quantified among YFP^+^ CD4 T cells (CD8^-^) by ICS (mean ±SD, n = 3 biological replicates/group). **g**, Gating strategy for flow sorting of healthy donor T cells co-transduced with lentiviruses expressing anti-mesothelin CAR (M5) and ho2R-GFP/ho9R-GFP (left plots). Right plots indicate MFI of orthogonal IL-2Rβ in double-positive cells. Source data

To evaluate the translational potential for o9R signalling, we generated human orthogonal IL-2Rβ (ho2R) and human orthogonal chimeric IL-2Rβ–IL-9R (ho9R) (Supplementary Table 3). Human T cells were retrovirally transduced with vectors encoding either ho2R or ho9R (each containing YFP), as well as a vector encoding a TCR specific for NY-ESO-1 in the context of HLA*0201 (NYESO1-TCR clone 1G4) (ref. ^47^). NY-ESO-1 is a cancer-testis antigen that is overexpressed in synovial sarcoma, myxoid liposarcoma, melanoma and other tumours. Transduced and sorted T cells (Extended Data Fig. 10a) were exposed to MSA-bound human orthogonal IL-2 (MSA-hoIL-2, which contains amino acid substitutions SQVLKA at positions 15, 16, 19, 20, 22 and 23 relative to the native polypeptide; Supplementary Table 4) or wild-type IL-2 (MSA-hIL-2)^48,49^. Consistent with observations in the mouse system, signalling through ho9R activates pSTAT1, pSTAT3 and pSTAT5 signalling (Fig. 4a). Likewise, native IL-2-STAT5 signalling through the endogenous γ_c_ is not disrupted by orthogonal receptor expression (Extended Data Fig. 10b). Finally, MSA-hoIL-2 is highly orthogonal and does not activate untransduced cells; however, wild-type MSA-hIL-2 does activate the orthogonal receptor at high concentrations (Extended Data Fig. 10b).

Similar to the mouse system, despite the weaker proliferative signal of ho9R signalling (Fig. 4b), we observed an expansion of T_SCM_ (CD45RA^+^CD27^+^CCR7^+^CD95^+^) and central memory T (T_CM_; CD45RA^−^CD27^+^CCR7^+^CD95^+^) cells by enrichment and absolute quantity among ho9R–NYESO1-TCR T cells after two and six days in culture with MSA-hoIL-2 (Fig. 4c,d and Extended Data Fig. 10c,d,e). This difference in T cell phenotype between ho9R–NYESO1-TCR and ho2R–NYESO1-TCR T cells persisted even after four antigen-specific challenges with an HLA*0201^+^NY-ESO-1^+^ melanoma cell line, nRFP-M407, in the presence of MSA-hoIL-2 (Fig. 4e,f). Compared to ho2R–NYESO1 T cells, ho9R–NYESO1 T cells retained a greater frequency of CD45RA^+^CD27^+^ and T_SCM_ cells, and expressed higher levels of CD62L and CXCR3. Repetitively stimulated ho9R–NYESO1 T cells expressed more IFNγ, TNF and IL-2 and exhibited greater polyfunctionality when exposed to either activating CD3/CD28 beads or the cognate melanoma cell line nRFP-M407 (Fig. 4g and Extended Data Fig. 10f). In a model of continuous antigen exposure^50^ using human T cells that co-express a mesothelin-specific CAR (M5) and either ho2R or ho9R, signalling through ho9R resulted in superior tumour cell killing after repetitive tumour challenges, with an enrichment of CD45RA^+^CD27^+^ T cells with higher CCR7 expression, similar to ho9R–NYESO1 T cells (Fig. 4h and Extended Data Fig. 10g).

In conclusion, the orthogonal IL-2 cytokine-receptor platform enables the redirection of signalling through modular replacement of the orthogonal IL-2Rβ ICD with the ICDs of receptors for other γ_c_ cytokines. Notably, specific in vivo stimulation of o9R signalling in TCR- and CAR-based tumour-specific T cells by the orthogonal IL-2 ligand 3A10 results in improved anti-tumour activity in two solid tumour models that are refractory to immunotherapy, and retains robust activity in a more stringent setting without conditioning lymphodepletion. This benefit is mediated by leveraging the γ_c_ cytokines to reroute the IL-2 signalling message through the IL-9Rα ICD, which results in the concomitant activation of STAT1, STAT3 and STAT5. Conveyed in T cells, the signalling message of IL-9Rα results in a unique phenotype that merges beneficial functional characteristics of stem cell memory and effector T cells, to provide improved in vivo anti-tumour activity.

## Methods

### Protein production

DNA encoding mouse and human wild-type and orthogonal IL-2 and wild-type IL-9 was cloned into the insect expression vector pAcGP67-A, which includes a C-terminal 8×His tag for affinity purification. DNA encoding mouse serum albumin (MSA) was purchased from Integrated DNA Technologies (IDT) and cloned into pAcGP67-A as an N-terminal fusion. Insect expression DNA constructs were transfected into *Trichoplusia ni* (High Five) cells (Invitrogen) using the BaculoGold baculovirus expression system (BD Biosciences) for secretion and purified from the clarified supernatant by Ni-NTA followed by size-exclusion chromatography with a Superdex-200 column and formulated in sterile phosphate-buffered saline (PBS) for injection. Endotoxin was removed using the Proteus NoEndo HC Spin column kit following the manufacturer’s recommendations (VivaProducts) and endotoxin removal was confirmed using the Pierce LAL Chromogenic Endotoxin Quantification Kit (Thermo Fisher Scientific). Proteins were concentrated and stored at −80 °C until ready for use.

### Mammalian expression vectors

cDNA encoding mouse orthogonal IL-2Rβ and geneblock cDNA encoding mouse ICDs of IL-4R, IL-7R, IL-9R and IL-21R (IDT) were cloned into the retroviral vector pMSCV-MCS-IRES-YFP by PCR and isothermal assembly (ITA). Human orthogonal IL-2Rβ (ho2R) and chimeric orthogonal IL-2Rβ-ECD–IL-9R-ICD (ho9R) were similarly cloned into the pMSCV vector.

### Mice

Mice were housed in animal facilities approved by the Association for the Assessment and Accreditation of Laboratory Care and used under protocols approved by the Institutional Animal Care and Use Committee (IACUC) at the University of California, Los Angeles (UCLA), University of Pennsylvania and Stanford University. For experiments conducted at UCLA, C57BL/6J mice were bred and kept in the Radiation Oncology Vivarium; pmel-1 TCR/Thy1.1 transgenic mice (pmel mice) on a C57BL/6 background were obtained from The Jackson Laboratory. For experiments conducted at University of Pennsylvania, C57BL/6J and B6 CD45.1 ‘Pepboy’ mice were purchased from The Jackson Laboratory. For experiments conducted at Stanford University, C57BL/6J mice were purchased from The Jackson Laboratory.

### Cell lines and cell culture

The B16-F10 mouse melanoma cell line was purchased from ATCC and cultured with RPMI 1640 with l-glutamine (Thermo Fisher Scientific) containing 10% fetal bovine serum (FBS, Omega Scientific), penicillin (100 U ml^−1^, Omega Scientific), streptomycin (100 μg ml^−1^, Omega Scientific) and amphotericin B (0.25 μg ml^−1^, Omega Scientific). The mouse pancreatic cancer cell line, derived from spontaneous tumours arising in KPC (*LSL-Kras*^*G12D/+*^*;LSL-Trp53*^*R172H/+*^*;Pdx-1-Cre*) mice, was maintained in RPMI 1640 medium supplemented with 10% FBS and 1% penicillin–streptomycin. Human melanoma cell lines M407 and M263 were established from patient biopsies under UCLA IRB approval 11–003254 and maintained in RPMI 1640 medium supplemented with 10% FBS and 1% penicillin–streptomycin; M407 cells were stably transduced to express nuclear RFP (nRFP) for use in a live-cell imaging assay^51^. T cells derived from C57BL/6 or pmel transgenic mice were cultured in RPMI 1640 with l-glutamine supplemented with 10% Hyclone FBS (Cytiva), antibiotics, 50 μM 2-mercaptoethanol (Gibco), 1% non-essential amino acids, 1% sodium pyruvate and HEPES. Primary human T cells were cultured similarly except without 2-mercaptoethanol. HEK293T cells were purchased from ATCC and maintained in DMEM supplemented with 10% FBS, 1× GlutaMax (Gibco) and penicillin–streptomycin. Cell lines were periodically authenticated and also periodically tested for mycoplasma infection using a mycoplasma detection kit (Biotool).

### Retrovirus production

Production of retroviruses encoding orthogonal cytokine receptors has been previously described^9^. In brief, HEK293T cells were seeded at 3 × 10^6^ cells per 10-cm tissue culture dish and allowed to adhere overnight. Cells were transfected with a 1.5:1 ratio of pMSCV retroviral vector to a pCL-Eco packaging vector using X-tremeGENE HP (Roche), Turbofect (Thermo Fisher Scientific) or TransIT Reagent (Thermo Fisher Scientific) and cultured overnight in DMEM with 5% FBS. After 24 h, the medium was replaced with fresh DMEM with 5% FBS and cultured for an additional 24 h. The medium was collected, clarified through centrifugation and flash-frozen in liquid nitrogen for storage at −80 °C. Cells were replenished with fresh DMEM with 5% FBS, and cultured for an additional 24 h, and retroviral supernatant was collected and stored as described. To generate retrovirus for the transduction of pmel and human T cells, the same procedure was used with the following variations: 18 h after transfection, the medium was replaced with DMEM with 10% FBS containing 20 mM HEPES and 10 mM sodium butyrate and incubated for 8 h. The medium was then replaced with DMEM with 10% FBS containing 20 mM HEPES and no sodium butyrate and incubated overnight. The next day, the medium was collected and filtered through a 0.45μm filter. If not used immediately, virus was frozen at −80 °C for later use. Retroviruses encoding chimeric antigen receptors were produced as previously described^52^. In brief, Plat-E packaging cells (Cell Biolabs) were transfected with pMSGV vectors using Lipofectamine 2000 (Thermo Fisher Scientific). Culture medium was replaced 24 h later and after an additional 24 h the medium was collected, clarified by centrifugation and passed through a 0.45-μm filter before storage at −80 °C.

### Adenovirus construction and purification

Replication-deficient E1/E3-deleted adenovirus vectors Ad5-CMV-oIL-2 (Ad-oIL-2) and Ad5-CMV-Null (Ad-Null) were constructed using the AdEasy XL Adenoviral Vector System (Agilent). Mouse orthogonal IL-2 clone 3A10 cDNA was synthesized (GenScript) with 5′ KpnI and 3′ HindIII restriction sites and subcloned into the multiple cloning site of pShuttle-CMV (Addgene). pAdEasy-1-containing BJ5183-AD-1 *Escherichia coli* cells were transformed with PmeI-linearized pShuttle-CMV-oIL-2 for homologous recombination. The resulting recombinant Ad plasmid was sequence-verified and expanded in XL10-Gold Ultracompetent cells before PacI linearization and transfection into HEK293T cells. High-titre adenoviruses were purified by caesium chloride (CsCl_2_) gradient centrifugation after multiple rounds of amplification. CsCl_2_ was exchanged to A195 buffer^53^ with Amicon Ultra-15 centrifugal filter units (Millipore). Viral titre (VP per ml) was determined spectrophotometrically (Nanodrop, Thermo Fisher Scientific).

### Lentivirus production

Lentiviral vectors for human T cell transduction were produced in HEK293T cells. Lentiviral plasmids for anti-human anti-mesothelin M5 CAR (previously described^50^) and human orthogonal receptors (ho2R or ho9R linked to GFP by a 2A sequence) together with packaging plasmids were transfected into HEK293T cells using Lipofectamine 3000 (Thermo Fisher Scientific). Ultracentrifugation was performed to concentrate lentiviral supernatants collected at 24 h or 48 h after transfection, and concentrated viruses were stored at −80 °C.

### Activation, retroviral transduction and sorting of primary mouse T cells

Viral transduction of C57BL/6-derived mouse T cells was previously described^9^. In brief, 12-well tissue culture plates were coated overnight with 2.5 μg ml^−1^ solution of anti-mouse CD3ε (clone 145-2C11, Biolegend) in sterile PBS. Single-cell suspensions were prepared from spleens and lymph nodes of 6–8-week-old C57BL/6J mice by dissociation through a 70-μm cell strainer followed by RBC lysis in ACK lysis buffer (Gibco). Cells were resuspended in mouse T cell medium containing 100 IU ml^−1^ recombinant mouse IL-2 (mIL-2) and activated with plate-bound anti-mouse CD3ε and soluble anti-mouse CD28 (5 μg ml^−1^, clone 37.51, BioXCell) for 24 h. Activated mouse T cells were transduced by spinfection using retroviral supernatants containing polybrene (10 μg ml^−1^) and 100 IU ml^−1^ mIL-2 at 2,600 rpm for 90 min at 32 °C. Viral supernatant was replaced with fresh mouse T cell medium containing 100 IU ml^−1^ mIL-2 and cultured for 24 h. Cells were collected via gentle pipetting and resuspended at 1 × 10^6^ per ml in fresh T cell medium containing 100 IU ml^−1^ mIL-2 and expanded overnight at 37 °C before further downstream cellular assays.

For retroviral transduction of pmel T cells, splenocytes from the five- to ten-week-old pmel mice were collected one to three days before transduction and activated with 50 U ml^−1^ mIL-2 (Peprotech) and 1 μg ml^−1^ mouse gp100 peptide (Anaspec). One day before transduction, six-well tissue culture plates were coated with Retronectin (Takara) and placed in a 4 °C refrigerator overnight. The following day, plates were blocked with 0.5% FBS in PBS for 30 min and washed with PBS. Viral supernatant (2 ml) was added to each well and spun at 2,000*g* for two hours. Activated pmel T cells (3 × 10^6^) were added to each well with 50 U ml^−1^ mouse IL-2 and spun at 2,000*g* for 10 min and then cultured at 37 °C for 18–24 h. Then, viable transduced cells were sorted based on expression of YFP and exclusion of 7-AAD using an Aria II cell sorter (BD Biosciences), and rested overnight before use in downstream in vitro or in vivo assays. Retroviral transduction of mouse CAR T cells was previously described^52^. In brief, primary donor CD45.1 mouse splenocytes (from 4–6-week-old female CD45.1 B6.SJL-Ptprca Pepcb/BoyJ mice) were enriched for CD3^+^ cells by magnetic bead separation (STEMCELL Technologies). T cells were activated with mouse CD3/CD28 Dynabeads (Thermo Fisher Scientific) in the presence of 50 U ml^−1^ recombinant human IL-2 (Peprotech) for 48 h before spinfection on retronectin-coated (Takara Bio) plates. Cell were collected for in vitro assays or intravenous injection two days after spinfection.

### Activation, retroviral transduction and sorting of primary human T cells

Primary human peripheral blood mononuclear cells (PBMCs) isolated from a healthy human donor by leukapheresis were thawed and rested overnight before activation for two days with anti-human CD3/28 magnetic Dynabeads (Thermo Fisher Scientific) and human IL-2 (500 U ml^−1^). T cells were co-transduced for 48 h on 6-well plates coated with Retronectin (Takara) and loaded with 1 ml per well of each retrovirus (encoding ho2R and NYESO1-TCR clone 1G4 or ho9R and NYESO1-TCR clone 1G4) by spinfection. Activated and transduced cells were collected and beads were removed by placing on an EasySep cell separation magnet for two minutes. Cells were stained with anti-human Vβ13.1 PE antibody (Beckman Coulter, recognizes the β-chain of the NYESO1-TCR clone 1G4), and 7-AAD live/dead dye before cell sorting based on the expression of YFP, Vβ13.1, and exclusion of 7-AAD using an Aria II cell sorter (BD Biosciences).

For transduction of human T cells with anti-mesothelin M5 CAR and human ortho-receptors, freshly isolated CD4^+^ and CD8^+^ T cells were mixed in a 1:1 ratio and activated with CD3/CD28 magnetic Dynabeads at a 3:1 bead-to-cell ratio. T cells were co-transduced 24 h later with lentiviral vectors encoding M5 CAR and ho2R-GFP or ho9R-GFP. On day 5, beads were removed from the culture. T cells were maintained at 0.8 × 10^6^ per ml until reaching resting state as determined by cell size using Multisizer 4 Coulter Counter (Beckman), then cryopreserved. For flow sorting of M5 CAR–ho2R-GFP and M5 CAR–ho9R-GFP double-positive T cells, the co-transduced T cells were thawed and rested overnight before staining with anti-human IgG F(ab’)2 (Jackson ImmunoResearch) for M5 CAR and LIVE/DEAD Aqua for dead-cell exclusion. GFP served as surrogate marker for ho2R and ho9R. Sorting was performed on a BD FACSAria Fusion (BD Biosciences).

### Phosphoflow signalling assay

Actively growing primary mouse or human T cells were rested in RPMI-C lacking IL-2 for 24 h before signalling assays. Cells were plated in an ultra-low-binding 96-well round bottom plate in 50 μl warm RPMI-C medium. Cells were stimulated by addition of recombinant cytokines for 20 min at 37 °C, and the reaction was terminated by fixation with 1.5% paraformaldehyde (PFA) for 15 min at room temperature with agitation. Cells were washed and permeabilized with ice-cold 100% methanol for 60 min on ice or stored at −80 °C overnight. Cells were washed with FACS buffer before staining with pSTAT antibodies (Supplementary Table 5) for 1 h at 4 °C in the dark. Cells were washed and analysed on a CytoFlex (Beckman Coulter). Data represent the mean fluorescence intensity (MFI), and points were fit to a log(agonist) versus dose–response model using Prism 8.4 (GraphPad). For the gating strategy, see Supplementary Fig. 2.

### Western blot

IL-2 and FBS-starved CAR T cells were stimulated for 20 min with cytokines and lysed with ice-cold RIPA buffer supplemented with protease/phosphatase inhibitor cocktail (Halt, Thermo Fisher Scientific) to extract protein. Thirty micrograms of total protein was loaded into SDS–PAGE gels (NuPage Bis-Tris, Thermo Fisher Scientific) and subsequently transferred to PVDF membranes (Immobilon-FL, Millipore). Detection of pSTAT1, pSTAT3, pSTAT5 and GAPDH was performed with respective primary antibodies followed by IRDye-labelled secondary antibodies or HRP-linked secondary antibodies. Membranes were imaged on an Odyssey CLx (LI-COR Biosciences).

### Mouse T cell proliferation assay

Actively growing primary mouse T cells were rested in RPMI-C lacking IL-2 for 48 h before labelling with CellTracer Violet (CTV, Thermo Fisher Scientific). Labelled cells were seeded at 50,000 T cells per well in 50 μl in a 96-well round-bottom plate. Cells were cultured for two days in serial dilutions of MSA-oIL-2. Cytokine was replenished on day 2. On day 4, CTV-labelled cell proliferation was evaluated by fluorescence-activated cell sorting (FACS) using the CytoFlex. Live-cell gates were based on FSC and SSC. CAR T cell proliferation was assessed by seeding 50,000 cells per well in a round-bottom 96-well plate in the presence of MSA-oIL-2 or MSA-IL-2. On day 2, cells were fed with fresh medium and cytokines. Daily cell counts were acquired by staining an aliquot of cells with Calcein AM viability dye (Thermo Fisher Scientific) and analysed on the Celigo Image Cytometer (Nexcelom Bioscience).

### Cytokine assays

For Luminex assays, transduced CAR T cells were incubated in round-bottom 96-well plates (50,000 cells per well) in triplicates for four days in the presence of cytokines after which supernatants were analysed with a Th1/Th2/Th9/Th17/Th22/Treg Cytokine 17-Plex Mouse ProcartaPlex Panel (Thermo Fisher Scientific). A cytokine bead array (BD Biosciences) was used to individually measure IFNγ from supernatant of B16-F10 coculture with pmel T cells according to the manufacturer’s instructions. oIL-2 expression from PDA7940b cells (10,000 cells per well, 96-well plate) was evaluated by mouse IL-2 ELISA (Abcam) in cell culture supernatants at various time points after infection with Ad-Null or Ad-oIL-2 (100 VP per cell). In vivo expression was assessed by injecting PBS, Ad-Null or Ad-oIL-2 (1 × 10^9^ VP per tumour) into PDA7940b tumours and collecting 72 h later. Tumours were dissociated by three freeze-thaw cycles and homogenates were analysed for mouse IL-2 by ELISA. Terminal blood was collected by cardiac puncture and processed to serum by centrifugation. IL-2 concentrations were normalized to total protein content.

### Real-time cell killing assays

PDA7940b tumour cells were seeded at 10,000 cells per well in a 96-well xCELLigence E-Plate (Agilent). Twenty-four hours later, transduced CAR T cells pre-incubated for 48 h in the presence of oIL-2 were added at a 2:1 ratio and the target cell index was recorded every 15 min in the Real-Time Cell Analysis (RTCA) Analyzer (Agilent). T cell killing of B16-F10 cell lines transduced with a nuclear localizing RFP was previously described^54^. In brief, B16-F10-RFP^+^ cells pulsed with 100 ng ml^−1^ IFNγ for 18 h were plated in a flat-bottom 96-well plate in triplicate at 5,000 cells per well for IncuCyte Live Cell Analysis (Essen Bioscience). Pmel T cells (o2R or o9R, pre-treated with MSA-IL-2 or MSA-oIL-2 for 48 h) were added at a 2:1 E:T ratio and two phase-contrast and fluorescent images were obtained of each well every two hours using the IncuCyte live imaging system and quantified by percentage confluence. The human TCR T cell repetitive killing assay was also conducted using IncuCyte Live Cell Analysis. Human melanoma cells (nRFP-M407, 5 × 10^5^) were plated per well in 6-well plates. Untransduced or co-transduced human T cells (co-transduced with either ho2R–NYESO1-TCR or ho9R–NYESO1-TCR, and pre-incubated for 48 h with MSA-hoIL-2) were added in duplicate at a 1:1 E:T ratio. Every 72 h, melanoma cells (nRFP-M407, 5 × 10^5^) were added to each well; orthogonal cytokine (MSA-hoIL-2) was replenished 24 h before every tumour rechallenge.

The human CAR T cell repetitive killing assay was conducted using the xCELLigence Analyzer. AsPC-1 human pancreatic tumour cells were seeded on a 96-well xCELLigence E-Plate at 10,000 cells per well. Twenty-four hours later, M5 CAR–ho2R or M5 CAR–ho9R cells pre-incubated for 48 h with MSA-hoIL-2 (1 µM) were added in triplicate at a 1:1 E:T ratio. Every 48 h, the CAR T cells were collected, washed, resuspended in fresh MSA-hoIL-2 and added on new wells of the E-Plate seeded with tumour cells (10,000 per well) the day before. After the last round of restimulation, the T cells were collected for phenotyping by flow cytometry.

### In vivo tumour studies

For in vivo B16-F10 tumour growth experiments, early-passage cell lines were used (fewer than 10 passages). B16-F10 cells (5 × 10^5^) were injected subcutaneously in the right flank of 6–10-week-old female C57BL/6 mice. Where indicated, mice were lymphodepleted with 500 cGy of total body irradiation one day before ACT. T cells (derived from female mice) were adoptively transferred approximately seven days after tumour inoculation, or when tumours became palpable. Specifically, 5 × 10^6^ sorted T cells were resuspended in 50 μl of PBS per mouse and administered through retroorbital injection. Where indicated, mice received treatment with cytokines: mouse serum albumin (MSA)-bound mouse IL-2 (MSA-IL-2) or MSA orthogonal IL-2 (MSA-oIL-2) (2.5 × 10^4^ units per day, intraperitoneal) for five consecutive days (or longer, where indicated) starting on the day of ACT. Tumour size (length × width) was monitored with calipers three times a week and volume was calculated as (length × width^2^)/2). Peripheral blood (10 μl) was collected at specified time points from the tail vein for quantification of adoptively transferred pmel T cells by flow cytometry. Mice were euthanized when the total tumour volume exceeded 2,000 mm^3^, as per IACUC guidelines.

The syngeneic PDA tumour model has been previously described^52^. In brief, PDA7940b tumours established subcutaneously in female C57BL/6 mice were treated intratumorally with control virus Ad-Null (1 × 10^9^ VP per injection) or Ad-oIL-2 (1 × 10^9^ VP per injection) in 50 μl PBS on days 0 and 4. CAR T cells (5 × 10^6^ live CAR-positive cells) were administered through tail-vein injection on day 1 in 200 μl PBS. Cyclophosphamide-based conditioning chemotherapy was performed on day −1 by intraperitoneal injection (120 mg kg^−1^). Tumour dimensions were measured with digital calipers and volumes were calculated as follows: volume = (length x width^2^)/2. Cured mice were rechallenged with PDA7940b cells by subcutaneous injection into the opposite flank and tumour size was recorded 24 days later by caliper measurement. Age-matched naive mice were injected identically and served as a control for tumour growth.

### Immunophenotyping by flow and mass cytometry

For in vitro immunophenotyping of orthogonal-cytokne-receptor-transduced T cells, sorted T cells were plated with equipotent doses of MSA-oIL-2 or MSA-IL-2 in triplicates. After 48 h, T cells were collected and surface-stained. For in vivo assessments, peripheral blood was obtained by tail-vein sampling at indicated time points. At the time of necropsy, spleens were crushed and washed with PBS over a 70-μm cell strainer to collect splenocytes. Splenocytes and peripheral blood samples were treated with ACK lysis buffer before antibody staining.

B16 tumours were minced and dissociated using a mouse tumour dissociation kit (Miltenyi Biotec) and a gentleMACS Octo Dissociator (Miltenyi Biotec). Cells were then resuspended in PBS and filtered through a 70-μm cell strainer to obtain single-cell suspensions. Cells were stained with antibodies at 4 °C for 30 min in FACS buffer. Antibodies are listed in Supplementary Table 5. The 7-AAD viability dye was used to distinguish live cells from dead cells. Cells were analysed by flow cytometry using a LSRFortessa (BD Biosciences) and data were collected using BD FACSDiva (v.6.1.2). Data were analysed using FlowJo software (v.10, BD Biosciences). PDA7940b tumours were excised, weighed, minced with scalpels and dissociated using an enzyme cocktail consisting of hyaluronidase (2.5 U ml^−1^), DNAse (50 U ml^−1^), collagen type I/II/IV (75 U ml^−1^, 35 U ml^−1^, 75 U ml^−1^, respectively) in RPMI 1640 supplemented with 1% penicillin–streptomycin. CD45-positive cells were isolated from single-cell tumour suspensions with CD45 (TIL) MicroBeads according to the manufacturer’s instructions (Miltenyi Biotec) and stored in liquid nitrogen. Quantification of tumour-infiltrating CAR T cells was performed using CountBright Beads (Thermo Fisher Scientific) and normalized to tumour weight. For gating strategy, see Supplementary Fig. 3.

For mass cytometry, cells were first fixed with 1.6% PFA for five minutes at room temperature. Cells were washed with 10 ml MaxPar Cell Staining Buffer (Fluidigm) and spun at 970*g* at 4 °C for 10 min. Next, the cells were resuspended in the surface antibody cocktail for 30 min at room temperature. Cells were washed with 5 ml of PBS and resuspended in 1 ml of ice-cold methanol for 15 min on ice. Cells were again washed with MaxPar Cell Staining Buffer and stained with the intracellular antibody cocktail for 30 min at room temperature. Finally, cells were washed with 10 ml MaxPar Cell Staining Buffer and stained with the intercalating solution (Cell-ID Intercalator-Ir, 201192B) at a 1:6,000 dilution in Maxpar Fix and Perm Buffer with 1.6% PFA (Fluidigm, 201067) overnight at 4 °C. Data were acquired using the Fluidigm Helios mass cytometer. Analysis was performed using Omiq based on arcsinh-scaled data gated on live, singlet CD45^+^ leukocytes or CD8^+^ T cells (for gating strategy, see Supplementary Fig. 4). Cells were embedded in two-dimensional visualization using opt-SNE and clustered using FlowSOM with elbow metaclustering using Euclidean distances. Differentially abundant clusters were determined using edgeR with a *P* value significance threshold of 0.05 and log-transformed fold change ≥ 1. Graphs were generated using the R package ggplot.

### Intracellular cytokine staining

Human co-transduced T cells (either ho2R–NYESO1-TCR or ho9R–NYESO1-TCR) were collected from repetitive tumour challenge coculture 72 h after the most recent tumour challenge and 24 h after orthogonal cytokine had been replenished in the culture medium. T cells (1 × 10^5^) were cultured in a 96-well plate with anti-human CD3/CD28 Dynabeads (Thermo Fisher Scientific) or melanoma cells at a 1:1 E:T ratio (nRFP-M407 or M263) in the presence of brefeldin A and monensin. After four hours, cells were surface stained for 30 min at room temperature, fixed and permeabilized for intracellular cytokine staining for 30 min at room temperature. For intracellular cytokine staining of enriched CD45^+^ tumour-infiltrating leukocytes from PDA740b tumours, cells were stimulated for six hours with Cell Activation Cocktail (with Brefeldin A) (Biolegend), fixed/permeabilized in the Cyto-Fast Fix/Perm Buffer Set (Biolegend) and stained with an anti-IFNγ antibody. Cells were washed and analysed by flow cytometry using a LSRII (BD Biosciences). Data were analysed using FlowJo software (v.10, BD Biosciences).

### Multiplex immunohistochemistry

Formalin-fixed and paraffin-embedded tumour specimens were cut in 4-μm-thick sections onto glass slides for staining. The tyramide signal amplification (TSA)‐based Opal method was used in this study for immunofluorescence staining (Opal Polaris 7‐Color Automation IHC Kit; Akoya Biosciences; NEL871001KT). The Opal fluorophores were used at a 1 in 150 dilution, as per the manufacturer’s recommendation. A fluorescent single-plex was performed for each biomarker and compared to the appropriate chromogenic single-plex to assess staining performance. Once each target was optimized with single-plex staining, the Opal 6 multiplexed assay was used to perform multiplex staining of slides. We applied primary antibodies to mouse spleen specimens as controls at optimized concentrations previously determined for single-plex staining of control tissues. Staining was performed using the BOND RX system (Leica Biosystems). The sequence of antibodies for multiplex staining was: FOXP3 (Opal 480), CD4 (Opal 520), PD-1 (Opal570), CD8 (Opal 620) and CD3 (Opal 690). Staining was performed after 20 min of heat-induced antigen retrieval using Bond Epitope Retrieval Solution 2 (Leica Biosystems). Antibodies are listed in Supplementary Table 5, and were used at a 1:200 dilution with a one-hour incubation. All fluorescently labelled slides were counterstained with DAPI and scanned on the Vectra Polaris (Akoya Biosciences) at ×20 magnification using appropriate exposure times. The data from the multispectral camera were analysed by the imaging InForm software (Akoya Biosciences) and quantification was performed using HALO image analysis software (Indica Labs).

### Histopathology, clinical chemistry and RNA ISH

Mice were euthanized by means of CO_2_ asphyxiation. Immediately after death, blood (*n* = 3 mice per group) was collected by cardiac puncture into Microvette tubes (Sarstedt) and allowed to clot at room temperature for 30 min before centrifugation at 12,000*g* for 10 min. Serum was stored at −80 °C before analysis. Cytokine levels in sera were measured with the Mouse 25-plex Cytokine Panel (IDEXX Bioanalytics). Serum levels of Ca, P, K and uric acid were measured with a custom clinical chemistry panel (IDEXX Bioanalytics).

Complete necropsy with macroscopic post-mortem examination was performed on all mice. Formalin-fixed tissues samples were trimmed according to the RITA guidelines (https://reni.item.fraunhofer.de/reni/trimming/) and then routinely processed for paraffin embedding, sectioning and haematoxylin and eosin (H&E) staining. The resulting slides were analysed by a board-certified veterinary pathologist blinded to experimental design.

The distribution of mesothelin expression and CAR T cell infiltration in the meninges were investigated by means of multiplex fluorescent RNA ISH (RNAscope Multiplex Fluorescent Assay, ACD Bio) including a custom probe designed against the mouse retrovirus used to transduce T cells with CAR and ortho-receptors. The assay was performed on formalin-fixed and paraffin-embedded brain sections. Whole-slide imaging on the resulting fluorescently labelled sections was performed using the Aperio VERSA 200 slide scanner (Leica Biosystems). CAR T cells and mesothelin-positive cells were finally counted using the object counting tool included in the Aperio ImageScope software (Leica Biosystems).

### RNA-seq and analysis

For the in vitro experiments described in Fig. 2 and related supplementary material, o2R and o9R pmel T cells were stimulated with 5 μM oIL-2 or 0.05 μM oIL-2 for 48 h. RNA was extracted using the RNeasy mini kit (Qiagen). RNA-seq libraries were prepared using the KAPA mRNA stranded library preparation kit, according to the manufacturer’s recommendations. Libraries were pooled and sequenced on the Illumina HiSeq3000 platform (50-bp single-end reads). Reads were aligned to the mouse reference genome (mm9/GRCm38) using HISAT2 (v.2.0.4) (ref. ^55^). Gene expression was quantified using HTSeq-counts (v.0.6.1) (ref. ^56^). Differential expression analysis was performed using DESeq2 (ref. ^57^), and subsequent gene set enrichment analysis was performed using the fgsea (ref. ^58^) and msigdbr (ref. ^59^) R packages, specifically on the TFactS annotated gene set^60^, and visualized using the ggplot2 R package. Differentially expressed genes were filtered to those with an adjusted *P* value of less than 0.01 and a log_2_-transformed fold change ≥ 1. Gene expression was visualized using the normalized gene expression (calculated using the rlog transform from DESeq2 and scaled by row) using the pheatmap R package. Principal component analysis and sample-to-sample heatmaps were generated using the R functions prcomp and dist, respectively.

For the in vitro experiments described in Fig. 3, CAR-o2R and CAR-o9R cells were stimulated for 48 h with MSA-oIL-2 or MSA-IL-2 and total RNA was extracted using the RNeasy mini kit (Qiagen). RNA expression was analysed using the nCounter Mouse Immunology Panel (Nanostring Technologies). Analysis was performed as described above.

### Statistics and reproducibility

All unpaired *t*-tests are two-sided. Exact *P* values are provided in Supplementary Table 6. For box and whisker plots (Fig. 2j), box plots represent median and first and third quartiles, and whiskers extend to minima and maxima. Unless otherwise stated, *n* refers to biological and not technical replicates. For mouse tumour growth and survival experiments, sample size was selected on the basis of previous work using the respective ACT models (B16-pmel and PDA7940b-mesothelin CAR). Mice with tumours of equal size were randomized before treatment; tumours were measured in a blinded fashion. Data in Fig. 1b–f are representative of three independent experiments. Data in Fig. 2b,e,g are representative of two independent experiments. In Fig. 2b, *n* = 6 mice per group except *n* = 5 for pmel + MSA-IL-2 and *n* = 4 for pmel + MSA-IL-2, lymphodepleted. In Fig. 2c, *n* = 6 mice per group, except *n* = 5 for BL6 T cells + MSA-IL-2 and pmel + MSA-IL-2. In Fig. 2d, *n* = 6 mice per group, except *n* = 5 for pmel + MSA-IL-2 and pmel + MSA-IL-2 (lymphodepleted), and *n* = 3 for BL6 T cells + MSA-IL-2. Data in Fig. 2c,d are representative of three independent experiments. Data in Fig. 2f are representative of two independent experiments and conclusions are confirmed by three independent experiments using flow cytometry (Fig. 2f), mass cytometry (Fig. 2h) and immunofluorescence (Extended Data Fig. 5g). Data in Fig. 3a,c–e are representative of two independent experiments. Data in Fig. 3b are representative of three independent experiments. Efficacy experiments (Fig. 3i,j) are representative of two independent experiments. Data in Fig. 4a,e–h are representative of two independent experiments. Data in Fig. 4b–d are representative of three independent experiments.

### Reporting summary

Further information on research design is available in the Nature Research Reporting Summary linked to this paper.

## Online content

Any methods, additional references, Nature Research reporting summaries, source data, extended data, supplementary information, acknowledgements, peer review information; details of author contributions and competing interests; and statements of data and code availability are available at 10.1038/s41586-022-04801-2.

## Supplementary information

Supplementary Information This file includes six supplementary tables (Supplementary Tables 1–6) and four Supplementary Figures (Supplementary Figures 1–4). Supplementary Tables 1 and 3 include the protein sequences of the mouse and human orthogonal and orthogonal chimeric receptors, respectively. Supplementary Tables 2 and 4 include the protein sequence of the orthogonal mouse and human IL2, respectively. Supplementary Table 5 includes a list of reagents used in the manuscript. Supplementary Table 6 is a list of exact P values from the manuscript. Supplementary Figure 1 is a full scan of the western blot images summarized in Fig. 3b, along with a figure legend. Supplementary Figure 2 is the flow cytometry gating strategy used for data in Fig. 1 along with a figure legend. Supplementary Figure 3 is the flow cytometry gating strategy used in Fig. 3k, along with a figure legend. Supplementary Figure 4 is the gating strategy used for mass cytometry data in Fig. 2h and Extended Data Fig. 5e-f, along with a figure legend.

Reporting Summary

Figures 1-4, Extended Data Figs 1–3, 5, 7, 9, 10.

## Data Availability

All data associated with this study are present in the manuscript or its Supplementary Information files. Gene expression data are available at https://www.ncbi.nlm.nih.gov/geo/ under accession numbers GSE199909 and GSE199956.
