# Publisher Correction: Potentiating adoptive cell therapy using synthetic IL-9 receptors

**DOI:** 10.1038/s41586-022-05548-6

**Published:** 2022-11-15

**Authors:** Anusha Kalbasi, Mikko Siurala, Leon L. Su, Mito Tariveranmoshabad, Lora K. Picton, Pranali Ravikumar, Peng Li, Jian-Xin Lin, Helena Escuin-Ordinas, Tong Da, Sarah V. Kremer, Amy L. Sun, Sofia Castelli, Sangya Agarwal, John Scholler, Decheng Song, Philipp C. Rommel, Enrico Radaelli, Regina M. Young, Warren J. Leonard, Antoni Ribas, Carl H. June, K. Christopher Garcia

**Affiliations:** 1 Department of Radiation Oncology, David Geffen School of Medicine and Jonsson Comprehensive Cancer Center, University of California, Los Angeles, Los Angeles, CA USA; 2 Parker Institute for Cancer Immunotherapy, San Francisco, CA USA; 3 Center for Cellular Immunotherapies, Perelman School of Medicine, University of Pennsylvania, Philadelphia, PA USA; 4 Departments of Molecular and Cellular Physiology and Structural Biology, Stanford University School of Medicine, Stanford, CA USA; 5 Laboratory of Molecular Immunology and the Immunology Center, National Heart, Lung, and Blood Institute, National Institutes of Health, Bethesda, MD USA; 6 Division of Hematology/Oncology, Department of Medicine, David Geffen School of Medicine and Jonsson Comprehensive Cancer Center, University of California, Los Angeles, Los Angeles, CA USA; 7 Penn Vet Comparative Pathology Core, Department of Pathobiology, University of Pennsylvania, Philadelphia, PA USA; 8 Stanford Cancer Institute, Stanford University School of Medicine, Stanford, CA USA; 9 Howard Hughes Medical Institute, Stanford University School of Medicine, Stanford, CA USA

**Keywords:** Synthetic biology, Immunotherapy, Cancer therapy, Interleukins

Correction to: *Nature* 10.1038/s41586-022-04801-2 Published online 8 June 2022

This paper was originally published under a standard Springer Nature license (© The Author(s), under exclusive licence to Springer Nature Limited). It is now available as an open-access paper under a Creative Commons Attribution 4.0 International license, © The Author(s). The error has been corrected in the HTML and PDF versions of the article.

