# Supplementary material for: Potentiating adoptive cell therapy using synthetic IL-9 receptors
Source: Nature. 2022 Jun 8;607(7918):360–5. doi: 10.1038/s41586-022-04801-2 (PMC9283313; doi:10.1038/s41586-022-04801-2)
Supplement: Supplementary file 1 — This file includes six supplementary tables (Supplementary Tables 1–6) and four Supplementary Figures (Supplementary Figures 1–4). Supplementary Tables 1 and 3 include the protein sequences of the mouse and human orthogonal and orthogonal chimeric receptors, respectively. Supplementary Tables 2 and 4 include the protein sequence of the orthogonal mouse and human IL2, respectively. Supplementary Table 5 includes a list of reagents used in the manuscript. Supplementary Table 6 is a list of exact P values from the manuscript. Supplementary Figure 1 is a full scan of the western blot images summarized in Fig. 3b, along with a figure legend. Supplementary Figure 2 is the flow cytometry gating strategy used for data in Fig. 1 along with a figure legend. Supplementary Figure 3 is the flow cytometry gating strategy used in Fig. 3k, along with a figure legend. Supplementary Figure 4 is the gating strategy used for mass cytometry data in Fig. 2h and Extended Data Fig. 5e-f, along with a figure legend. [file 41586_2022_4801_MOESM1_ESM.pdf]

nature portfolio

https://doi.org/10.1038/s41586-022-04801-2

Supplementary information

Potentiating adoptive cell therapy using synthetic IL-9 receptors

In the format provided by the
authors and unedited

Supplementary Table 1. Sequences of Mouse Chimeric Orthogonal Cytokine Receptors.

Protein sequence of chimeric orthogonal mouse receptors. Transmembrane regions underlined; chimeric receptor ICD regions highlighted in purple.

Receptor	Sequence
Orthogonal mouse IL-2 Receptor (o2R)	MATIALPWSLSLYVFLLLLATPWASAAVKNCSHLECFYNSRANVSCMWSHEEALNVTTCHVHAKSNLRHWNKTCE<br>LTLVRQASWACNLILGSFPESQSLTSVDLLDINVVCWEEKGWRRVKTCDFHPFDNLRLVAPHSLQVLHIDTQRCNIS<br>WKVSQVSDFIEPYLEFEARRRLLGHSWEDASVLSLKQRQQWLFLEMLIPSTSYEVQVRVKAQRNNTGTWSPWSQ<br>PLTFRTRPADPMKEILPMSWLRYLLLVLGCFSGFFSCVYILVKCRYLGPWLKTVLKCHIPDPSEFFSQLSSQHGGDL<br>QKWLSSPVPLSFFSPSGPAPEISPLEVLDGDSKAVQLLLLQKDSAPLPSPSGHSQASCFTNQGYFFFHLPNALEIES<br>CQVYFTYDPCVEEEVEEDGSRLPEGSPHPPLLPLAGEQDDYCAFPPRDDLLLFSPSLSTPNTAYGGSRAPEERSPL<br>SLHEGLPSLASRDLMGLQRPLERMPEGDGEGLSANSSGEQASVPEGNLHGQDQDRGQ<br>GPILTLNTDAYLSLQELQAQDSVHLI*
Chimeric Orthogonal mouse IL-4 Receptor (o4R)	MATIALPWSLSLYVFLLLLATPWASAAVKNCSHLECFYNSRANVSCMWSHEEALNVTTCHVHAKSNLRHWNKTCE<br>LTLVRQASWACNLILGSFPESQSLTSVDLLDINVVCWEEKGWRRVKTCDFHPFDNLRLVAPHSLQVLHIDTQRCNIS<br>WKVSQVSDFIEPYLEFEARRRLLGHSWEDASVLSLKQRQQWLFLEMLIPSTSYEVQVRVKAQRNNTGTWSPWSQ<br>PLTFRTRPAFQLPLIQRLPLGVTISCLCIPLFCLFCYFSITKIKKIWWDQIPTPARSPLVAIIIQDAQVPLWDKQTRSQES<br>TKYPHWKTCLDKLLPCLLKHRVKKKTDFPKAAPTKSLQSPGKAGWCPMEVSRTVLWPENVSVSVVRCMELFEAPV<br>QNVEEEEDEIVKEDLSMSPENSGGCGFQESQADIMARLTENLFSDLLEAENGGLGQSALAESCSPLPSGSGQASV<br>SWACLPMGPSEEATCQVTEQPSHPGPLSGSPAQSAPTLACTQVPLVLADNPAYRSFSDCCSPAPNPGELAPEQQ<br>QADHLEEEEPPSPADPHSSGPPMQPVESWEQILHMSVLQHGAAAGSTPAPAGGYQEFVQAVKQGAAQDPGVPG<br>VRPSGDPGYKAFSSLLSSNGIRGDTAAAGTDDGHGGYKPFQNPVPNQSPSSVPLFTFGLDTELSPSPLNSDPPKS<br>PPECLGLELGLKGGDWVKAPPPADQVPKPFGDDLGFGIVYSSLTCHLCGHLKQHHSQEEGGQSPIVASPGCGCC<br>YDDRSPSLGSLSGALESCPEGIPPEANLMSAPKTPSNLSGEGKGPGHSPVPSQTTEVPVGALGIAVS*
Chimeric Orthogonal mouse IL-7 Receptor (o7R)	MATIALPWSLSLYVFLLLLATPWASAAVKNCSHLECFYNSRANVSCMWSHEEALNVTTCHVHAKSNLRHWNKTCE<br>LTLVRQASWACNLILGSFPESQSLTSVDLLDINVVCWEEKGWRRVKTCDFHPFDNLRLVAPHSLQVLHIDTQRCNIS<br>WKVSQVSDFIEPYLEFEARRRLLGHSWEDASVLSLKQRQQWLFLEMLIPSTSYEVQVRVKAQRNNTGTWSPWSQ<br>PLTFRTRPAKNQGGWDPVLPSVTILSLFSVFLLVILAHVLWKKRIKPVVWPSLPDHKKTLEQLCKKPKTSLNVSFNPE<br>SFLDCQIHEVKGVEARDEVESFLPNDLPAQPEELETQGHRAAVHSANRSPETSVSPPETVRRESPLRCLARNLSTC<br>NAPPLLSSRSPDYRDGDRNRPPVYQDLPNSGNTLNVPVPVPQPLPFQSGILIPVSQRQPISTSSVLNQEEAYVTMS<br>SFYQNK*
Chimeric Orthogonal mouse IL-9 Receptor (o9R)	MATIALPWSLSLYVFLLLLATPWASAAVKNCSHLECFYNSRANVSCMWSHEEALNVTTCHVHAKSNLRHWNKTCE<br>LTLVRQASWACNLILGSFPESQSLTSVDLLDINVVCWEEKGWRRVKTCDFHPFDNLRLVAPHSLQVLHIDTQRCNIS<br>WKVSQVSDFIEPYLEFEARRRLLGHSWEDASVLSLKQRQQWLFLEMLIPSTSYEVQVRVKAQRNNTGTWSPWSQ<br>PLTFRTRPAQRRQGLLVPRWQWSASILVVVPIFLLLTGFVHLLFKLSPRLKRIFYQNIPSPEAFFHPLYSVYHGDFQS<br>WTGARRAGPQARQNGVSTSSAGSESSIWEAVATLTYSPACPVQFACLKWEATAPGFPGLPGSEHVLPAGCLELE<br>GQPSAYLPQEDWAPLGSARPPPPDSDSGSSDYCMLDCCEECHLSAFPGHTESPELTLAQPVALPVSSRA*
Chimeric Orthogonal mouse IL-21 Receptor (o21R)	MATIALPWSLSLYVFLLLLATPWASAAVKNCSHLECFYNSRANVSCMWSHEEALNVTTCHVHAKSNLRHWNKTCE<br>LTLVRQASWACNLILGSFPESQSLTSVDLLDINVVCWEEKGWRRVKTCDFHPFDNLRLVAPHSLQVLHIDTQRCNIS<br>WKVSQVSDFIEPYLEFEARRRLLGHSWEDASVLSLKQRQQWLFLEMLIPSTSYEVQVRVKAQRNNTGTWSPWSQ<br>PLTFRTRPAGEPEAGWDPHMLLLLAVLIIVLVFMGLKIHLPWRLWKKIWAPVPTPESFFQPLYREHSGNFKKWVNTP<br>FTASSIELVPQSSTTTSALHLSLYPAKEKKFPGLPGLEEQLECDGMSEPGHWCIIPLAAGQAVSAYSEERDRPYGLV<br>SIDTVTVGDAEGLCVWPCSCEDDGYPAMNLDAGRESGPNSEDLLLVTDPAFLSCGCVSGSGLRLGGSPGSLLDRL<br>RLSFAKEGDWTADPTWRTGSPGGGSESEAGSPPGLDMDTFDSGFAGSDCGSPVETDEGPPRSYLRQWVVRTPP<br>PVDSGAQSS*

Supplementary Table 2. Protein sequence of mouse MSA-orthogonal IL2 Cytokine. MSA sequence underlined. Cytokine sequence highlighted in purple.

**Cytokine**	**Sequence**
Mouse serum albumin (MSA)-Orthogonal mouse IL-2 (3A10) – 8xHis	MLLVNQSHQGFNKEHTSKMVSAIVLYVLLAAAAHSAFAGSRGVFRREAHKSEIAHRYNDLGEQHFKGLVLIAFS<br>QYLQKCSYDEHAKLVQEVTDFAKTCVADESAANCDKSLHTLFGDKLCAIPNLRENYGELADCCTKQEPERNEC<br>FLQHKDDNPSLPPFERPEAEAMCTSFKENPTTFMGHYLHEVARRHPYFYAPELLYYAEQYNEILTQCCAEADK<br>ESCLTPKLDGVKEKALVSSVRQRMKCSSMQKFGERAFKAWAVARLSQTFPNADFAEITKLATDLTKVNKECCH<br>GDLLECADDRAELAKYMCENQATISSKLQTCCDKPLLKKAHCLSEVEHDTMPADLPAIAADFVEDQEVCKNYA<br>EAKDVFLGTFLYEYSRRHPDYSVSLLLRLAKKYEATLEKCCAEANPPACYGTVLAEFQPLVEEPKNLVKTNCDL<br>YEKLGEYGFQNAILVRYTQKAPQVSTPTLVEAARNLGRVGTKCCTLPEDQRLPCVEDYLSAILNRVCLLHEKTP<br>VSEHVTKCCSGSLVERRPCFSALTVDETYVPKEFKAETFTFHSDICTLPEKEKQIKKQTALAELVKHKPKATAEQ<br>LKTVMDDFAQFLDTCCKAADKDTCFSTEGPNLVTRCKDALAGGGGSAPTSSSTSSSTAEAQQQQQQQQQQQ<br>QHLDNLLVLLKALLSRMENYRNLKLPRMLTFKFYLPKQATELKDLQCLEDELGPLRHVLDLTQSKSFQLEDAEN<br>FISNIRVTVVKLKGSDNTFECQFDDESATVVDFLRRWIAFCQSIISTSPQAAAHHHHHHHH*

Supplementary Table 3. Sequences of Human Orthogonal Cytokine Receptors. Protein sequence of human orthogonal IL-2Rβ (o2R, top) and chimeric orthogonal IL2Rβ–ECD/IL9R-ICD (o9R, bottom). Transmembrane region underlined; IL-9R ICD region highlighted in purple.

**Receptor**	**Sequence**
Orthogonal human IL-2 Receptor (o2R)	MAAPALSWRLPLLILLLPLATSWASAAVNGTSQFTCFYNSRANISCVWSQDGALQDTSCQVHAWPDR<br>RRWNQTCELLPVSQASWACNLILGAPDSQKLTTVDIVTLRVLCREGVRWRVMAIQDFKPFENLRLMAPI<br>SLQVVHVETHRCNISWEISQASDFFERHLEFEARTLSPGHTWEEAPLLTLKQKQEWICLETLTPDTQYE<br>FQVRVKPLQGEFTTWSPWSQPLAFRTKPAALGKDTIPWLGHLLVGLSGAFGFIILVYLLINCRNTGPWL<br>KKVLKCNTPDPSKFFSQLSSEHGGDVQKWLSSPFPSSSFSPGGLAPEISPLEVLERDKVTQLLLQQDK<br>VPEPASLSSNHSLTSCFTNQGYFFFHLPDALEIEACQVYFTYDPYSEEDPDEGVAGAPTGSSPQPLQP<br>LSGEDDAYCTFPSRDDLLLFSPSLLGGPSPPSTAPGGSGAGEERMPPSLQERVPRDWDPQPLGPPTP<br>GVPDLVDFQPPPELVLREAGEEVPDAGPREGVSFPWSRPPGQGEFRALNARLPLNTDAYLSLQELQG<br>QDPTHLV*
Chimeric Orthogonal human IL-9 Receptor (o9R)	MAAPALSWRLPLLILLLPLATSWASAAVNGTSQFTCFYNSRANISCVWSQDGALQDTSCQVHAWPDR<br>RRWNQTCELLPVSQASWACNLILGAPDSQKLTTVDIVTLRVLCREGVRWRVMAIQDFKPFENLRLMAPI<br>SLQVVHVETHRCNISWEISQASDFFERHLEFEARTLSPGHTWEEAPLLTLKQKQEWICLETLTPDTQYE<br>FQVRVKPLQGEFTTWSPWSQPLAFRTKPAQRQGPLIPPWGWPGNTLVAVSIFLLLTGPTYLLFKLSPR<br>VKRIFYQNVPSPAMFFQPLYSVHNGNFQTWMGAHGAGVLLSQDCAGTPQGALEPCVQEATALLTCGP<br>ARPWKSVALEEEQEGPGTRLPGNLSSEDVLPAGCTEWRVQTLAYLPQEDWAPTSLTRPAPPDSEGS<br>RSSSSSSSSNNNNYCALGCYGGWHLSALPGNTQSSGPIPALACGLSCDHQGLETQQGVAWVLAGHC<br>QRPGLHEDLQGMLLPSVLSKARSWTF*

Supplementary Table 4. Protein sequence of human MSA-orthogonal IL2 Cytokine. MSA sequence underlined. Cytokine sequence highlighted in purple.

Cytokine	Sequence
Mouse serum albumin (MSA)- Orthogonal human IL-2 (SQVLKA) – 8xHis	MLLVNQSHQGFNKEHTSKMVSAIVLYVLLAAAAHSAFAGSRGVFRREAHKSEIAHRYNDL<br>GEQHFKGLVLIAFSQYLQKCSYDEHAKLVQEVTDFAKTCVADESAANCDKSLHTLFGDKLC<br>AIPNLRENYGELADCCTKQEPERNECFLQHKDDNPSLPPFERPEAEAMCTSFKENPTTFM<br>GHYLHEVARRHPYFYAPELLYYAEQYNEILTQCCAEADKESCLTPKLDGVKEKALVSSVRQ<br>RMKCSSMQKFGERAFKAWAVARLSQTFPNADFAEITKLATDLTKVNKECCHGDLLECADD<br>RAELAKYMCENQATISSKLQTCCDKPLLKKAHCLSEVEHDTMPADLPAIAADFVEDQEVCK<br>NYAEAKDVFLGTFLYEYSRRHPDYSVSLLLRLAKKYEATLEKCCAEANPPACYGTVLAEFQ<br>PLVEEPKNLVKTNCDLYEKLGEYGFQNAILVRYTQKAPQVSTPTLVEAARNLGRVGTKCCT<br>LPEDQRLPCVEDYLSAILNRVCLLHEKTPVSEHVTKCCSGSLVERRPCFSALTVDETYVPK<br>EFKAETFTFHSDICTLPEKEKQIKKQTALAELVKHKPKATAEQLKTVMDDFAQFLDTCCKAA<br>DKDTCFSTEGPNLVTRCKDALAGGGGSPGAPTSSSTKKTQLQLSQLLVLLKAILNGINNYK<br>NPKLTRMLTFKFYMPKKATELKHLQCLEEELKPLEEVLNLAQSKNFHLRPRDLISNINVIVLE<br>LKGSETTFMCEYADETATIVEFLNRWITFCQSIISTLTAAAHHHHHHHH*

Supplementary Table 5. Reagents.

Type	Reagent	Company	Catalog #
Antibody	Anti-mouse CD45 (89Y), clone 30-F11	Biolegend	103120
Antibody	Anti-mouse CD11c (209Bi), clone N418	Biolegend	117302
Antibody	Anti-mouse CD69 (143Nd), clone H1.2F23	Biolegend	104502
Antibody	Anti-mouse F4/80 (146Nd), clone BM8	DVS	31460008B
Antibody	Anti-mouse CD11b (148Nd), clone M1/70	Biolegend	101214
Antibody	Anti-mouse CD19 (149Nd), clone 6D5	Biolegend	115514
Antibody	Anti-mouse Ly6C (150Nd or 162Dy), clone HK1.4	Biolegend	128002
Antibody	Anti-mouse Ly6G (151Eu), clone 1A8	Biolegend	127602
Antibody	Anti-mouse CD3e (152Sm), clone 145-2C11	Biolegend	100314
Antibody	Anti-mouse CD28, clone 37.41	BioXCell	BE0015-1
Antibody	Anti-mouse CD274 (153Eu), clone 10F.9G2	DVS	3153016B
Antibody	Anti-mouse CD25 (150Nd or 155Gd or 151Eu), clone 3C7	Biolegend	101906
Antibody	Anti-mouse CD279 (159Tb), clone 29F.1A12	Biolegend	135202
Antibody	Anti-mouse CD335 (167Er), clone 29A1.4	DVS	3167008B
Antibody	Anti-mouse CD8a (168Er), clone 53-6.7	Biolegend	100716
Antibody	Anti-mouse CD161 (170Er), clone D13.14.4E	Biolegend	108712
Antibody	Anti-mouse CD44 (171Yb), clone IM7	Biolegend	103014
Antibody	Anti-mouse CD40, clone HM40-3, LEAF Purifiied	Biolegend	HM40-3
Antibody	Anti-mouse CD4 (112Cd), clone RM4-5	Biolegend	100561
Antibody	Anti-mouse MHC II (IA/IE) (174Yb), clone M5/114.15	Biolegend	107610
Antibody	Anti-mouse CD103 (175Lu or 155Gd), clone 2E7	Biolegend	121402
Antibody	Anti-mouse CD45R/B220 (144Nd), clone RA3-6B2	DVS	3176002B
Antibody	Anti-mouse CD27 (139La), clone LG.3A10	Biolegend	124202
Antibody	Anti-mouse CD39 (142^{Nd}), clone 24DMS1	DVS	3142005B
Antibody	Anti-mouse CD69 (143Nd), clone H1.2F3	DVS	3143004B
Antibody	Anti-mouse CD62L (160Gd), clone MEL-14	DVS	3160008B
Antibody	Anti-mouse CD197 (164Dy), clone 4B12	DVS	3164013A
Antibody	Anti-mouse Ly-6A/E (169Tm), clone D7	DVS	3169015B
Antibody	Anti-mouse CD127 (175Lu), clone A7R34	Biolegend	135029
Antibody	Anti-mouse CD278 (176Lu), clone 7E.17G9	DVS	3176014B
Antibody	Anti-mouse Ki67 (115In), clone SolA15	eBioscience	14-5698-82
Antibody	Anti-mouse TNFa (141Pr), clone MP6-XT22	DVS	3141013B
Antibody	Anti-mouse EOMES (147Sm), clone Dan11mag	ThermoFisher	14-4875-82
Antibody	Anti-mouse pSTAT5 (150Nd), clone 47	DVS	3150005A
Antibody	Anti-mouse pSTAT1 (153Eu), clone 58D6	DVS	3153003A
Antibody	Anti-mouse pSTAT3 (158Gd), clone 4/p-STAT3	DVS	3158005A
Antibody	Anti-mouse Tbet (161Dy), clone 4B10	DVS	3161014B
Antibody	Anti-mouse BCL-6 (163Dy), clone K112-91	DVS	3163012B
Antibody	Anti-mouse IFNg (165Ho), clone XMG1.2	DVS	3165003B
Antibody	Anti-mouse GATA3 (167Er), clone TWAJ1	DVS	3167007A
Antibody	Anti-mouse CD73 (154Sm), clone CD73	DVS	3154019B
Antibody	Anti-mouse Thy1.1 (162Dy), clone OX-7	Biolegend	202501
Antibody	Anti-mouse Foxp3 (158Gd), clone FJK-16s	DVS	3158003A
Antibody	Anti-mouse CD45 (BV510), clone 30F11	BD Biosciences	563891

Antibody	Anti-mouse CD8 (BV421), clone 53-6.7	Biolegend	100738
Antibody	Anti-mouse CD62L (PE-Cy7), clone MEL-14	ThermoFisher/eBioscience	25-0621-82
Antibody	Anti-mouse CD44 (APC-Cy7), clone IM7	BD Biosciences	560568
Antibody	Anti-mouse CD44 (FITC), clone IM7	Biolegend	103005
Antibody	Anti-mouse CD95 (BV605), clone SA367H8	Biolegend	152612
Antibody	Anti-mouse Thy1.1/CD90.1 (AF700), clone OX-7	Biolegend	202528
Antibody	Anti-mouse CD45.1 (PE), clone REA11	Miltenyi	130-121-214
Viability Stain	LIVE/DEAD™ Fixable Aqua Dead Cell Stain	ThermoFisher	L34957
Antibody	Anti-mouse IL9R (CD129) APC, clone S18011E	Biolegend	158705
Antibody	Anti-mouse IL2Rb (PE) (clone 5H4)	Biolegend	105906
Antibody	biotinylated $F(ab')_2$ –fragment specific IgG	Jackson ImmunoResearch	109-066-006
Other	Streptavidin BV785	Biolegend	405249
Other	CountBright™ Absolute Counting Beads	ThermoFisher	C36950
Viability Stain	7-AAD	Beckman Coulter	A07704
Antibody	Anti-mouse GAPDH (clone 14C10 or D16H11)	Cell Signaling Technologies	5174S
Antibody	Anti-pSTAT1 Tyr701, clone 58D6	Cell Signaling Technologies	9167S
Antibody	Anti-pSTAT1 Tyr701 (PE) clone 58D6	Cell Signaling Technologies	8062S
Antibody	Anti-pSTAT3 Tyr705, clone EP2147Y	Abcam	ab76315
Antibody	Anti-pSTAT3 pY705 (AF647), clone 4/P-STAT3	BD Biosciences	612599
Antibody	Anti-pSTAT5 Tyr694, clone C11C5	Cell Signaling Technologies	9359S
Antibody	Anti-pSTAT5 Tyr694, (AF647), clone 47/STAT5	BD Biosciences	612599
Cytometric Bead	Mouse IFNg Flex Set	BD Biosciences	558296
Antibody	IRDye® 800CW Goat anti-Rabbit IgG (H + L) Secondary	LI-COR Biosciences	926-32211
Antibody	IRDye® 680RD Goat anti-Rabbit IgG Secondary Antibody	LI-COR Biosciences	926-68071
Antibody	Anti-rabbit IgG, HRP-linked Antibody	Cell Signaling Technologies	7074S
Antibody	Anti-mouse CD3, Rabbit polyclonal – Opal 480	DAKO	A0452
Antibody	Anti-mouse CD4, Rabbit clone EPR19514 – Opal 520	Abcam	AB183685
Antibody	Anti-mouse CD8, Rat clone 4SM15 – Opal 570	Ebioscience	14-0808
Antibody	Anti-mouse PD-1, Rabbit – Opal 690	Abcam	Ab21442
Antibody	Anti-human Vβ13.1 (PE)	Beckman Coulter	IM2292
Antibody	Anti-human CD45RA (APC or BV421), clone HI100	Biolegend	304112
Antibody	Anti-human CD27 (PE-CF594 or APC), clone M-T271	Biolegend	562297
Antibody	Anti-human CD95 (PE-Cy7), clone DX2	Biolegend	305622
Antibody	Anti-human CCR7 (BV711), clone G043H7	Biolegend	353229
Antibody	Anti-human CD62L (BV650), clone DREG-56	Biolegend	304832
Antibody	Anti-human CXCR3 (PE-CF594), clone 1C6	BD Biosciences	560831
Antibody	Anti-human CD4 (BV510), clone OKT4	Biolegend	317444
Antibody	Anti-human CD8 (BV605), clone RPA-T8	Biolegend	301040
Antibody	Anti-human IFNγ (PE), clone 4S.B3	BD Biosciences	559326
Antibody	Anti-human TNFα (PerCP-Cy5.5), clone MAb11	Biolegend	502926
Antibody	Anti-human IL-2 (BV711), clone MQI-17H12	Biolegend	500346
Other	TransIT Transfection Reagent	Mirus	MIR2705
Other	Brefeldin A	Biolegend	420601
Other	Monensin	Biolegend	420701

Supplementary Table 6. Exact P Values.

Figure	Comparison	Exact p-value
1d	MSA-IL2 (50nM) o2R vs. MSA-oIL2 (5uM) o2R	0.1161
1d	MSA-IL2 (50nM) o4R vs. MSA-oIL2 (5uM) o4R	<0.0001
1d	MSA-IL2 (50nM) o7R vs. MSA-oIL2 (5uM) o7R	<0.0001
1d	MSA-IL2 (50nM) o9R vs. MSA-oIL2 (5uM) o9R	<0.0001
1d	MSA-IL2 (50nM) o21R vs. MSA-oIL2 (5uM) o21R	<0.0001
1e	MSA-IL2 (50nM) o2R vs. MSA-oIL2 (5uM) o2R	0.0242
1e	MSA-IL2 (50nM) o4R vs. MSA-oIL2 (5uM) o4R	0.0003
1e	MSA-IL2 (50nM) o7R vs. MSA-oIL2 (5uM) o7R	<0.0001
1e	MSA-IL2 (50nM) o9R vs. MSA-oIL2 (5uM) o9R	<0.0001
1e	MSA-IL2 (50nM) o21R vs. MSA-oIL2 (5uM) o21R	<0.0001
1f	MSA-IL2 (50nM) o2R vs. MSA-oIL2 (5uM) o2R	0.3366
1f	MSA-IL2 (50nM) o4R vs. MSA-oIL2 (5uM) o4R	<0.0001
1f	MSA-IL2 (50nM) o7R vs. MSA-oIL2 (5uM) o7R	0.9992
1f	MSA-IL2 (50nM) o9R vs. MSA-oIL2 (5uM) o9R	<0.0001
1f	MSA-IL2 (50nM) o21R vs. MSA-oIL2 (5uM) o21R	<0.0001
2b	pmel+MSA-IL2 (lymphodepleted) vs pmel + MSA-IL2	0.0011
2b	o2R pmel + MSA-IL2 vs o2R pmel + MSA-oIL2	0.0099
2b	o9R pmel + MSA-IL2 vs o9R pmel + MSA-oIL2	0.0037
2c	pmel+MSA-IL2 (lymphodepleted) vs pmel + MSA-IL2	0.0079
2d	pmel+MSA-IL2 (lymphodepleted) vs pmel + MSA-IL2	0.0074
2d	o9R pmel + MSA-IL2 vs o9R pmel + MSA-oIL2	0.0091
2d	o9R pmel + MSA-oIL2 vs pmel + MSA-IL2	0.0044
2e	pmel + MSA-IL2 5 days (lymphodepleted) vs o2R pmel + MSA-oIL2 5 days	0.0014
2e	pmel + MSA-IL2 5 days (lymphodepleted) vs pmel + MSA-IL2 5 days	0.009
2e	o9R pmel + MSA-IL2 25 days vs. pmel + MSA-IL2 5 days (lymphodepleted)	0.0063
2f	o9R pmel + MSA-oIL2 vs o2R pmel + MSA-oIL2	0.0292
2g	o9R pmel + MSA-oIL2 vs o2R pmel + MSA-oIL2	0.0044
2j	o9R pmel + MSA-oIL2 vs o2R pmel + MSA-oIL2	0.0033
3f	CAR-o2R + MSA-oIL2 vs CAR-o9R + MSA-oIL2 (IFNg)	<0.0001
3f	CAR-o2R + MSA-oIL2 vs CAR-o9R + MSA-oIL2 (TNFa)	<0.0001
3f	CAR-o2R + MSA-oIL2 vs CAR-o9R + MSA-oIL2 (IL-4)	<0.0001
3f	CAR-o2R + MSA-oIL2 vs CAR-o9R + MSA-oIL2 (IL-9)	<0.0001
3f	CAR-o2R + MSA-oIL2 vs CAR-o9R + MSA-oIL2 (IL-10)	<0.0001
3f	CAR-o2R + MSA-oIL2 vs CAR-o9R + MSA-oIL2 (IL-18)	<0.0001
3f	CAR-o2R + MSA-oIL2 vs CAR-o9R + MSA-oIL2 (IL-22)	<0.0001

3f	CAR-o2R + MSA-oIL2 vs CAR-o9R + MSA-oIL2 (IL-23)	<0.0001
3k	Ad-oIL2 + CAR vs Ad-oIL2 + CAR-o2R (left panel)	<0.0001
3k	Ad-oIL2 + CAR-o2R vs Ad-oIL2 + CAR-o9R (left panel)	0.0002
3k	Ad-oIL2 + CAR vs. Ad-oIL2 + CAR-o9R (right panel)	0.0447
3k	Ad-oIL2 + CAR-o2R vs. Ad-oIL2 + CAR-o9R (right panel)	0.0168
4b	ho9R/NYESO1-TCR + MSA-oIL2 vs ho2R/NYESO-1 TCR + MSA-oIL2	0.000939
4c	ho9R/NYESO1-TCR + MSA-oIL2 vs ho2R/NYESO-1 TCR + MSA-oIL2	p<0.0001
4d	ho9R/NYESO1-TCR + MSA-oIL2 vs ho2R/NYESO-1 TCR + MSA-oIL2 (Tscm)	0.01111
4d	ho9R/NYESO1-TCR + MSA-oIL2 vs ho2R/NYESO-1 TCR + MSA-oIL2 (Tcm)	0.00789
4h	ho9R/M5 CAR T cells + MSA-oIL2 vs ho2R/M5 CAR T cells + MSA-oIL2; 144-192 hrs	0.0392
4h	ho9R/M5 CAR T cells + MSA-oIL2 vs ho2R/M5 CAR T cells + MSA-oIL2; 192-240 hrs	0.0023
4h	ho9R/M5 CAR T cells + MSA-oIL2 vs ho2R/M5 CAR T cells + MSA-oIL2; CD45RA+CD27+	0.0013
4h	ho9R/M5 CAR T cells + MSA-oIL2 vs ho2R/M5 CAR T cells + MSA-oIL2; CCR7 MFI	0.0003

**Extended Data Fig.**	**Comparison**	**Exact p-value**
4a	o9R pmel + MSA-oIL2 vs o2R pmel + MSA-oIL2 (pSTAT5)	0.0012
4a	o9R pmel + MSA-oIL2 vs o2R pmel + MSA-oIL2 (pSTAT3)	0.0088
4a	o9R pmel + MSA-oIL2 vs o2R pmel + MSA-oIL2 (pSTAT1)	0.0047
4b	o9R pmel + MSA-oIL2 vs o2R pmel + MSA-oIL2	0.0122
5d	o9R pmel + MSA-IL2 25 days vs. o9R pmel + MSA-oIL2 5 days	0.0177
5d	o9R pmel + MSA-IL2 25 days vs. pmel + MSA-IL2 5 days (lymphodepleted)	0.0013
5d	o9R pmel + MSA-IL2 25 days vs. o2R pmel + MSA-oIL2 5 days	<0.0001
5i	o9R pmel + MSA-oIL2 vs o2R pmel + MSA-oIL2	0.0256
7d	MSA-oIL2+CAR-o2R vs. MSA-oIL2+CAR-o9R (Q1)	0.9813
7d	MSA-oIL2+CAR-o2R vs. MSA-oIL2+CAR-o9R (Q2)	<0.0001
7d	MSA-oIL2+CAR-o2R vs. MSA-oIL2+CAR-o9R (Q3)	<0.0001
7d	MSA-oIL2+CAR-o2R vs. MSA-oIL2+CAR-o9R (Q4)	<0.0001
8c	Ad-oIL2 + CAR-o9R vs. Ad-oIL2 + CAR-o2R	0.0212
8d	Ad-oIL2 + CAR-o9R vs. Ad-oIL2 + CAR-o2R	0.036
9a	Ad-oIL2 + CAR-o2R (No CTX) vs. Ad-oIL2 + CAR-o9R (No CTX)	0.0095
9a	Ad-oIL2 + CAR-o2R vs. Ad-oIL2 + CAR-o9R	<0.0001
10e	ho9R/NYESO1-TCR + MSA-oIL2 vs ho2R/NYESO-1 TCR + MSA-oIL2	0.0053

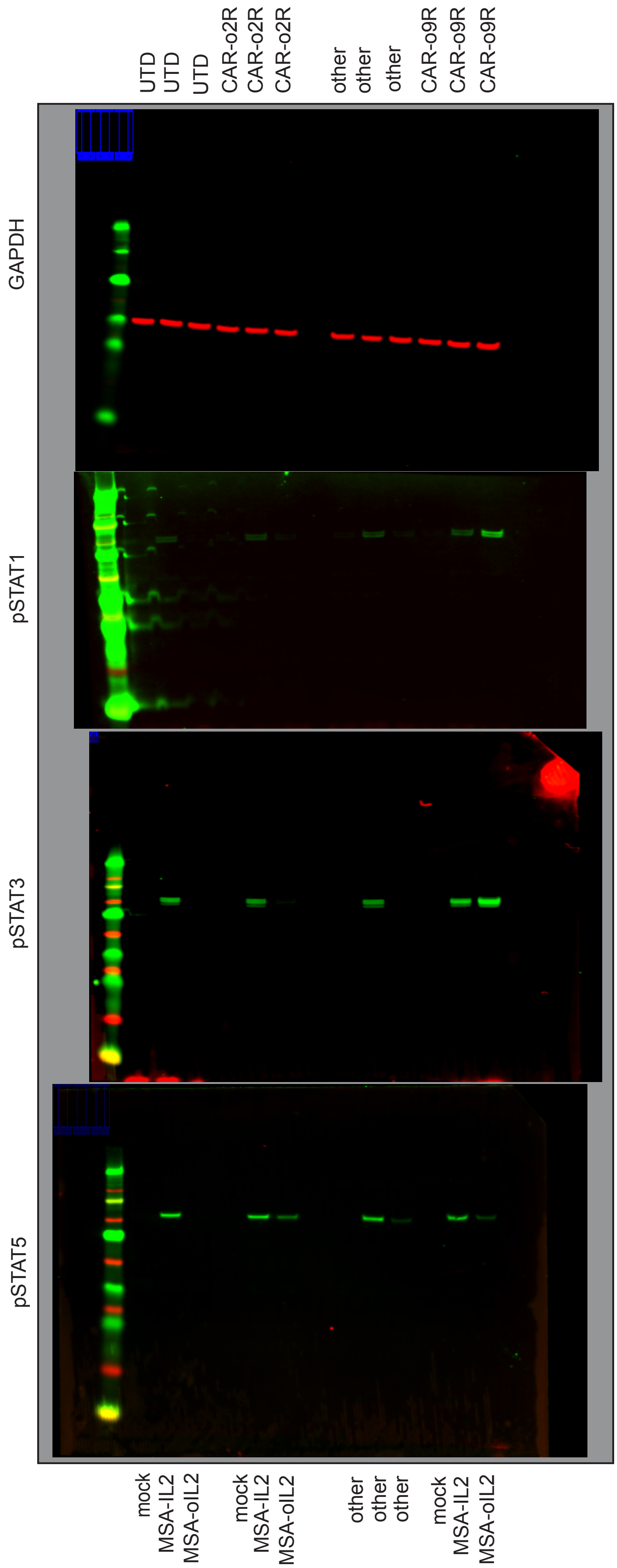

Supplementary Figure 1. Full-sized western blot images corresponding to Fig. 3b. Western blot analysis of GAPDH, pSTAT1, pSTAT3, pSTAT5 expression in T cells 30' after stimulation with MSA-IL2 (100nM) or MSA-oIL2 (5uM). Each gel includes a molecular weight ladder (far left lane). Sample identifiers are denoted at the top of the figure in line with the corresponding lane (UTD = untransduced, o2R = CAR-o2R, o9R = CAR-o9R, other = sample not related to Fig. 3b). Treatments for each sample are denoted below the figure in line with the corresponding lane.

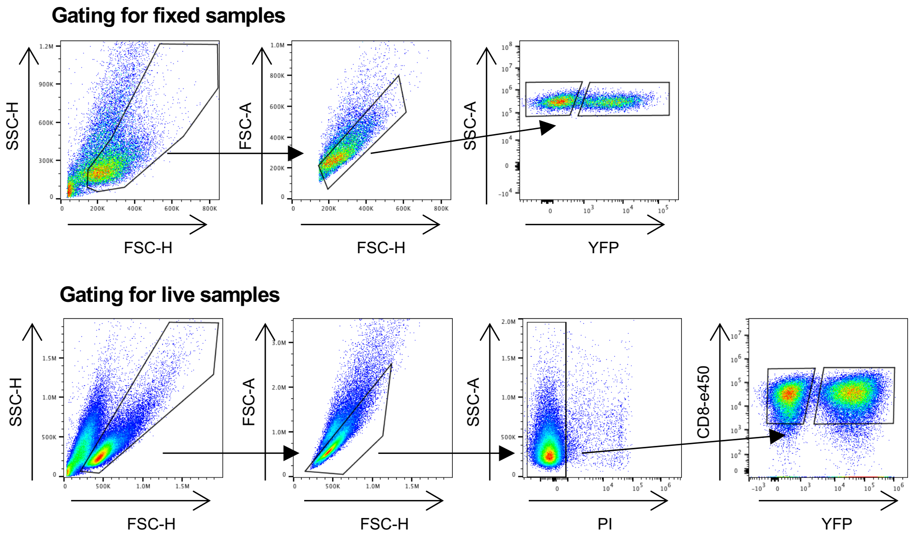

Supplementary Figure 2. Gating strategy for flow cytometry data in Fig. 1 to determine the intracellular expression pSTAT1, pSTAT3, pSTAT5, and pSTAT6 (fixed samples, top row) and surface expression of CD62L, Fas and Sca-1 (live samples, bottom row) among YFP+ mouse T cells.

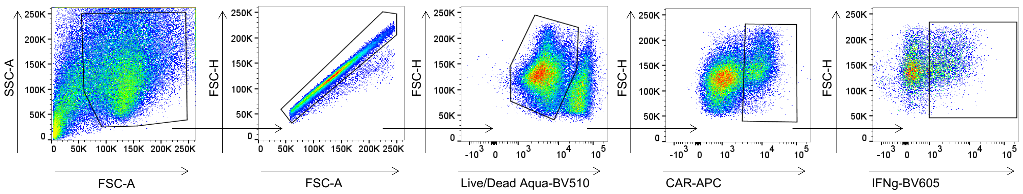

Supplementary Figure 3. a, Gating strategy for flow cytometry data in Fig. 3k to quantify live, singlet CAR+ tumor-infiltrating T cells and quanitfy intracellular IFNɣ.

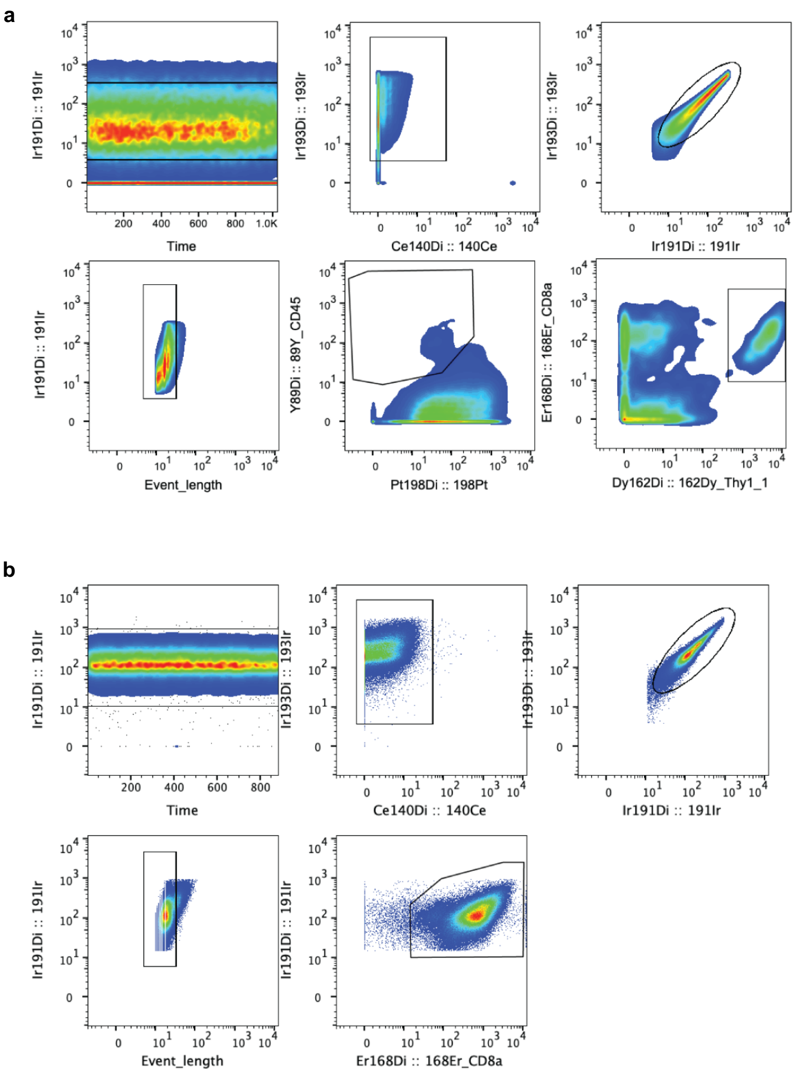

Supplementary Figure 4. a, Gating strategy for mass cytometry data in Fig. 2h to capture live, singlet CD8+ T cells from an in vitro T cell culture for downstream analysis of T cell phenotype and function. **b,** Gating strategy for mass cytometry data in Extended Data Fig. 5e-f to capture tumor-infiltrating live, singlet CD8+ T cells for downstream analysis of T cell quantity, phenotype and function.
