# Supplementary material for: Potentiating adoptive cell therapy using synthetic IL-9 receptors
Source: Nature. 2022 Jun 8;607(7918):360–5. doi: 10.1038/s41586-022-04801-2 (PMC9283313; doi:10.1038/s41586-022-04801-2)
Supplement: Supplementary file 2 — Reporting Summary [file 41586_2022_4801_MOESM2_ESM.pdf]

nature research

Corresponding author(s): Anusha Kalbasi, Antoni Ribas, Carl H. June, K. Christopher Garcia

Last updated by author(s): Mar 31, 2022

Reporting Summary

Nature Research wishes to improve the reproducibility of the work that we publish. This form provides structure for consistency and transparency in reporting. For further information on Nature Research policies, see our Editorial Policies and the Editorial Policy Checklist.

Statistics

For all statistical analyses, confirm that the following items are present in the figure legend, table legend, main text, or Methods section.

n/a	Confirmed	
☐	☒	The exact sample size (*n*) for each experimental group/condition, given as a discrete number and unit of measurement
☐	☒	A statement on whether measurements were taken from distinct samples or whether the same sample was measured repeatedly
☐	☒	The statistical test(s) used AND whether they are one- or two-sided<br>*Only common tests should be described solely by name; describe more complex techniques in the Methods section.*
☒	☐	A description of all covariates tested
☒	☐	A description of any assumptions or corrections, such as tests of normality and adjustment for multiple comparisons
☐	☒	A full description of the statistical parameters including central tendency (e.g. means) or other basic estimates (e.g. regression coefficient) AND variation (e.g. standard deviation) or associated estimates of uncertainty (e.g. confidence intervals)
☒	☐	For null hypothesis testing, the test statistic (e.g. *F*, *t*, *r*) with confidence intervals, effect sizes, degrees of freedom and *P* value noted<br>*Give P values as exact values whenever suitable.*
☒	☐	For Bayesian analysis, information on the choice of priors and Markov chain Monte Carlo settings
☒	☐	For hierarchical and complex designs, identification of the appropriate level for tests and full reporting of outcomes
☒	☐	Estimates of effect sizes (e.g. Cohen's *d*, Pearson's *r*), indicating how they were calculated

Our web collection on statistics for biologists contains articles on many of the points above.

Software and code

Policy information about availability of computer code

Data collection	BD FACSDiva (v6.1.2);
Data analysis	HISAT2 (v2.0.4); HTSeq-counts (v0.6.1); DESeq2 (v3.1.2); fgsea (v.3.12); msigdbr (v7.2.1); pheatmap (v1.0.12); bowtie2 (v.2.3.5); tophat2 (v2.1.1); edgeR (v3.12); Halo (v3.1); Omiq (no version available); GraphPad Prism (v9.0.0); FlowJo (v10.7.1)

For manuscripts utilizing custom algorithms or software that are central to the research but not yet described in published literature, software must be made available to editors and reviewers. We strongly encourage code deposition in a community repository (e.g. GitHub). See the Nature Research guidelines for submitting code & software for further information.

Data

Policy information about availability of data

All manuscripts must include a data availability statement. This statement should provide the following information, where applicable:

- Accession codes, unique identifiers, or web links for publicly available datasets
- A list of figures that have associated raw data
- A description of any restrictions on data availability

All data associated with this study are present in the manuscript or supplementary materials. Gene expression data are available at www.ncbi.nih.gov/geo/.

Field-specific reporting

Please select the one below that is the best fit for your research. If you are not sure, read the appropriate sections before making your selection.

☒ Life sciences ☐ Behavioural & social sciences ☐ Ecological, evolutionary & environmental sciences

For a reference copy of the document with all sections, see nature.com/documents/nr-reporting-summary-flat.pdf

Life sciences study design

All studies must disclose on these points even when the disclosure is negative.

Sample size	For mouse experiments in Figure 2 and 3, sample sizes were not determined using statistical methods, but based on extensive experience in the literature (including from our group) using the pmel/B16 and the PDA/mesoCAR mouse model.
Data exclusions	No data were excluded from the analysis.
Replication	For Figure 1, in vitro signaling experiments were repeated at least three times, with each data point in triplicates. In vitro proliferation experiments were repeated two times, with each data point in triplicate. For figure 2 and related supplementary material, in vitro experiments were repeated at least a total of two times, except for CyTOF, RNA-sequencing and Luminex cytokine analysis experiments. For CyTOF experiments, data was validated by flow cytometry experiments conducted in replicate experiments. For RNA-sequencing, biological triplicates were used. For luminex cytokine analysis, biological triplicates were used.
Randomization	For mouse experiments in Figure 2 and 3, mice were selected based on similar tumor size and groups were generated randomly.
Blinding	For mouse experiments in Figure 2 and 3, tumors were measured by an individual blinded to randomization.

Reporting for specific materials, systems and methods

We require information from authors about some types of materials, experimental systems and methods used in many studies. Here, indicate whether each material, system or method listed is relevant to your study. If you are not sure if a list item applies to your research, read the appropriate section before selecting a response.

Materials & experimental systems

n/a	Involved in the study	
☐	☒	Antibodies
☐	☒	Eukaryotic cell lines
☒	☐	Palaeontology and archaeology
☐	☒	Animals and other organisms
☒	☐	Human research participants
☒	☐	Clinical data
☒	☐	Dual use research of concern

Methods

n/a	Involved in the study	
☒	☐	ChIP-seq
☐	☒	Flow cytometry
☒	☐	MRI-based neuroimaging

Antibodies

Antibodies used	All antibodies used in the study, including clone and catalog number, are listed in Supplementary Table 5.
Validation	All antibodies used are commercially available and validation is noted on the manufacturer's website.

Eukaryotic cell lines

Policy information about cell lines

Cell line source(s)	B16-F10: ATCC; PDA7940b: Dr. Gregory Beatty, University of Pennsylvania; PLAT-E: Cell Biolabs; HEK293T: ATCC; M407 and M263: Dr. Antoni Ribas, UCLA;
Authentication	Cell lines were periodically authenticated (at least once per year) using short tandem repeat analysis, with the exception of HEK293T cells which were used at low passage after freezing aliquots upon receipt from ATCC.
Mycoplasma contamination	Cell lines were periodically tested (at least once per year) for mycoplasma infection using mycoplasma detection kit (Biotool).
Commonly misidentified lines (See ICLAC register)	No commonly misidentified lines were used.

Animals and other organisms

Policy information about studies involving animals; ARRIVE guidelines recommended for reporting animal research

Laboratory animals	(1) mouse, C57/BL6, female, 6-10 weeks of age; (2) mouse, pmel-1 transgenic (B6.Cg-Thy1a/Cy Tg(TcraTcrb)8Rest/J), female, 6-10 weeks of age, and (3) mouse, B6 CD45.1 (B6.SJL-Ptprca Pepcb/BoyJ), female 4-6 weeks of age.
Wild animals	The study did not involve wild animals.
Field-collected samples	The study did not involve samples collected from the field.
Ethics oversight	Mice were used under protocols approved by Institutional Animal Care and Use Committee at the University of California, Los Angeles (UCLA), University of Pennsylvania and Stanford University.

Note that full information on the approval of the study protocol must also be provided in the manuscript.

Flow Cytometry

Plots

Confirm that:

☒ The axis labels state the marker and fluorochrome used (e.g. CD4-FITC).

☒ The axis scales are clearly visible. Include numbers along axes only for bottom left plot of group (a 'group' is an analysis of identical markers).

☒ All plots are contour plots with outliers or pseudocolor plots.

☒ A numerical value for number of cells or percentage (with statistics) is provided.

Methodology

Sample preparation	Biological source: primary mouse cells in culture; single cell suspensions of mouse tumors after enzymatic digestion; single suspension of mouse spleen after mechanical dissociation, filtering and RBC lysis; single cell suspension of mouse lymph nodes after mechanical dissociation and filtering. Sample preparation: after exposure to experimental conditions, samples were washed with primary antibodies at 4C for 30 minutes in PBS with 5% fetal bovine serum and 2mM EDTA. Samples were washed and stained with 7-AAD or LiveDead Aqua viability dye to distinguish live and dead cells. For phosphoflow experiments, cells were washed and permeabilized with ice-cold 100% methanol for 60 minutes on ice or stored at -80C overnight. Cells were washed prior to staining with primary antibodies.
Instrument	Cells were analyzed by flow cytometry using a LSRFortessa (BD Biosciences) or a CytoFlex (Beckman Coulter).
Software	Data were collected using BD FACSDiva software and analyzed using FlowJo (v10.7.1).
Cell population abundance	For sorted o2R and o9R transduced pmel T cells (Figure 2 and related extended data), cells were sorted based on expression of YFP and consisted of 10-30% of live cells in the sorted sample. The purity of the sort was >90% as confirmed by expression of YFP in post-sort samples.
Gating strategy	For all analysis, dead cells and debris were gated out using FSC-A/SSC-A and viability staining. Singlets were gated based on FSC-A and FSC-H. For subsequent gating, some populations (e.g. CD45+ leukocytes, CD3+ T cells, etc.) were gated based on distinct expression of relevant markers. For populations without a clearly demarcated fluorescent signal, fluorescent-minus-one (FMO) and/or isotype staining were used to determine appropriate gating of positive and negative populations.

☒ Tick this box to confirm that a figure exemplifying the gating strategy is provided in the Supplementary Information.
